# Design, Synthesis, and Evaluation of Pyrrole-Based Selective MAO-B Inhibitors with Additional AChE Inhibitory and Neuroprotective Properties Identified via Virtual Screening

**DOI:** 10.3390/ph18111677

**Published:** 2025-11-05

**Authors:** Emilio Mateev, Samir Chtita, Ekaterina Pavlova, Ali Irfan, Diana Tzankova, Shubham Sharma, Borislav Georgiev, Alexandrina Mateeva, Georgi Momekov, Maya Georgieva, Alexander Zlatkov, Magdalena Kondeva-Burdina

**Affiliations:** 1 Department of Pharmaceutical Chemistry, Faculty of Pharmacy, Medical University—Sofia, 1000 Sofia, Bulgariaa.dineva@pharmfac.mu-sofia.bg (A.M.); mgeorgieva@pharmfac.mu-sofia.bg (M.G.); azlatkov@pharmfac.mu-sofia.bg (A.Z.); 2 Laboratory of Analytical and Molecular Chemistry, Faculty of Sciences Ben M’Sik, Hassan II University of Casablanca, Casablanca 20100, Morocco; samirchtita@gmail.com; 3 Department of Pharmacology, Pharmacotherapy and Toxicology, Faculty of Pharmacy, Medical University—Sofia, 1000 Sofia, Bulgaria; ekatherinevp@gmail.com (E.P.); gmomekov@pharmfac.mu-sofia.bg (G.M.); 4 Department of Chemistry, Government College University Faisalabad, Faisalabad 38000, Pakistan; raialiirfan@gmail.com; 5 Department of Chemistry, GLA University, Mathura 281406, Uttar Pradesh, India; shubham.sharma@gla.ac.in; 6 Institute of Biodiversity and Ecosystem Research, Bulgarian Academy of Sciences, 1113 Sofia, Bulgaria; bobogeorgiev5@gmail.com

**Keywords:** virtual screening, consensus docking, synthesis, multitarget ligands, neuroprotection, antioxidants

## Abstract

**Background:** Virtual screening is a widely adopted technique for the discovery of novel pharmacologically active compounds; however, the risk of identifying false positive hits remains a major challenge. **Aim:** The aim of this study was to perform a validated structure-based drug design screening to discover multitarget pyrrole-based molecules as selective dual-acting monoamine oxidase (MAO) and acetylcholinesterase (AChE) inhibitors. **Methods**: The study employed validated docking protocols using Glide (Schrödinger) and GOLD (CCDC), integrating ligand enrichment analysis and robust Molecular Mechanics/Generalized Born Surface Area (MM/GBSA) rescoring. These methods were applied to a custom-designed database of pyrrole-based compounds. The top-ranked hits were synthesized and validated through in vitro tests, demonstrating significant inhibitory activities against MAO-A, MAO-B, AChE, and Butyrylcholinesterase (BChE). **Results:** The docking protocols achieved favorable hit rates, with 25.93% for AChE inhibitors and 44.44% for MAO-B inhibitors. Additionally, structure–activity relationship analysis revealed key substituent effects that significantly influence binding affinity and selectivity. Two compounds, *EM-DC-19* (2-(2,5-dimethyl-1*H*-pyrrol-1-yl)-3-(2*H*-imidazol-4-yl)propanoic acid) and *EM-DC-27* ([4-(2,5-dimethyl-1*H*-pyrrol-1-yl)phenyl]acetic acid), were identified as selective MAO-B inhibitors with additional moderate AChE inhibitory activity, demonstrating IC_50_ values of 0.299 ± 0.10 µM and 0.344 ± 0.10 µM against MAO-B, and 76.15 ± 6.12 µM and 375.20 ± 52.99 µM against AChE, respectively. The absence of statistically significant inhibitory effects of these lead compounds on MAO-A and BChE (IC_50_ > 100 µM) underscores their selective inhibitory activity towards MAO-B and AChE. Furthermore, both compounds demonstrated low neurotoxicity and significant neuroprotective and antioxidant effects in rat brain synaptosomes, mitochondria, and microsomes. These effects were particularly evident in models of 6-hydroxydopamine-induced neurotoxicity (6-OHDA) and oxidative stress induced by tert-butyl hydroperoxide and Fe^2+^/ascorbic acid. **Conclusions**: The findings suggest that these multitarget compounds hold promise for further development, with potential for structural modifications to enhance their enzyme inhibitory and neuroprotective properties.

## 1. Introduction

Monoamine oxidase-B (MAO-B) inhibitors play a significant role in the management of Parkinson’s disease (PD), providing moderate symptomatic relief by increasing dopamine availability in the brain. These inhibitors are commonly used as monotherapy in the early stages of PD and as adjunct therapy with Levodopa (L-DOPA) in advanced stages to take care of the motor complications. Among currently approved drugs, rasagiline stands out for its potential disease-modifying effects, as supported by extensive long-term clinical data [1]. The therapeutic importance of MAO-B inhibition in PD was further reinforced by the approval of safinamide, a selective and reversible MAO-B inhibitor that also modulates glutamate release. Safinamide has demonstrated efficacy in improving both motor and non-motor symptoms of PD, including cognitive and emotional disturbances. Its approval by the European Medicines Agency (EMA) in 2015 and the U.S. Food and Drug Administration (FDA) in 2017 highlights the ongoing relevance and expanding therapeutic potential of MAO-B inhibitors in Parkinson’s disease management [2].

Parkinson’s disease is characterized not only by motor symptoms but also by a wide spectrum of non-motor symptoms, among which cognitive impairment is notably prevalent and profoundly impacts patients’ quality of life. It is increasingly recognized that motor and cognitive symptoms frequently co-occur in PD, reflecting the multifactorial neurodegenerative processes involving dopaminergic, cholinergic, and other neurotransmitter systems. This co-occurrence necessitates therapeutic strategies that can simultaneously target multiple pathways to improve both motor functions and cognitive deficits effectively. Recent papers highlight that dual inhibition of MAO-B and acetylcholinesterase (AChE) presents a promising approach to such multitarget treatments, as these enzymes play pivotal roles in modulating dopaminergic and cholinergic neurotransmission, respectively, both critically impaired in PD pathology [3,4].

While dopaminergic and MAO-B-targeted therapies primarily address the motor symptoms of PD, cognitive impairment remains a substantial non-motor challenge impacting patients’ quality of life. Therefore, cholinesterase inhibitors (ChEIs) such as rivastigmine, donepezil, and galanthamine have shown clinical effectiveness in improving cognitive function. The mode of action of ChEIs is the inhibition of enzymatic breakdown of acetyl- and/or butyrylcholinesterase (AChE, BChE), thereby enhancing cholinergic neurotransmission in the brain. Their benefits have been demonstrated in PD patients exhibiting dementia and in those with mild cognitive deficits. The therapeutic utility of ChEIs in PD highlights the importance of multitarget treatment strategies capable of simultaneously addressing both motor and cognitive symptoms, and demonstrates the growing interest in compounds that can modulate multiple cholinesterase enzymes alongside other relevant targets [5].

Virtual screening is a widely utilized computational technique designed to identify novel molecules that act as active enzyme inhibitors. Among the various structure-based drug design approaches, molecular docking stands out as the most commonly employed method. Docking significantly reduces the time and resources required for the identification and optimization of potential drug candidates by simulating the interaction between small molecules and the target protein. It predicts the best possible binding pose of a compound within the enzyme’s active site and calculates the binding energy of the resulting protein–ligand complex [6]. According to recent large-scale studies, molecular docking-based virtual screening campaigns exhibit a hit rate ranging from approximately 5% up to 35%, markedly outperforming traditional high-throughput experimental screening approaches [7]. These promising hit rates demonstrate docking’s increasing reliability and efficiency in drug discovery pipelines.

**Figure 1:**
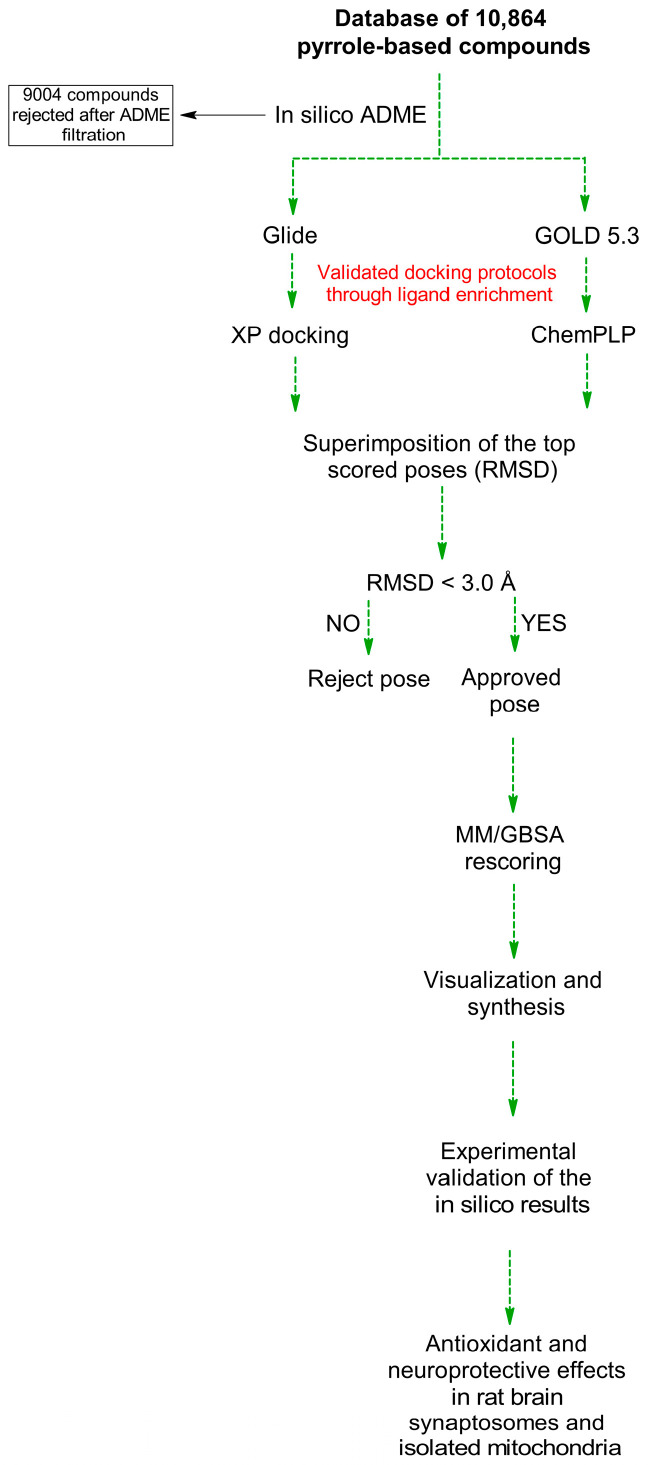
Overview of the study workflow. The schematic illustrates the major steps undertaken, including construction of a pyrrole-based compound library, initial ADME filtering, virtual screening using consensus docking protocols (Glide XP, MM-GBSA rescoring, and GOLD 5.3 validation), selection of top-ranked hits, synthesis of lead compounds, and subsequent in vitro evaluation of MAO-B and AChE inhibitory activities along with neuroprotective assessments.

This study focuses on utilizing multiple docking programs in a consensus docking approach to enhance overall enrichment and increase the number of true positive MAO-B and AChE inhibitors identified. Initially, the study involves thorough validation of the docking protocols for both enzymes through rigorous ligand enrichment analyses, ensuring the reliability and accuracy of the computational methods. Subsequently, a small database of pyrrole-based compounds is constructed and employed for virtual screening. The top-ranked hits from the docking studies are then synthesized and subjected to in vitro evaluation to determine their inhibitory activities against MAO-A, MAO-B, AChE, and BChE. Furthermore, the most promising multitarget ligands are assessed for their antioxidant and neuroprotective effects using rat brain mitochondria and synaptosomes as biological models. This integrative approach aims to identify potent multitarget compounds that can address key pathological features of neurodegeneration (Figure 1).

## 2. Results and Discussion

### 2.1. Validation of the Docking Protocols

Early investigations by our research groups focused on validating two of the most reliable docking software programs—GOLD 5.3 and Glide (Schrödinger Release 2025-4: Glide, Schrödinger, LLC, New York, NY, USA, 2025)—in the active sites of the enzymes MAO-B and AChE [8]. The results demonstrated that Glide has a slightly better ability to identify active inhibitors among the top ranks [9]. We identified the most optimal MAO-B X-ray structure as PDB **2V5Z** (with co-crystallized safinamide) and **4EY6** from the deposited AChE crystal structures [10]. However, several limitations were identified in our previous studies: water molecules were removed from the enzyme active sites during docking with GOLD 5.3; the more robust MM-GBSA rescoring method was not applied; the docking protocols utilized only a single docking software; and additional enrichment calculations (such as ROC, EF*, DEF, etc.) were not performed.

Therefore, this study focuses on utilizing several docking programs (consensus docking) to achieve better overall enrichment and thus increase the number of true positive MAO-B and AChE inhibitors in the structure-based virtual screening protocol. This will decrease the time needed to identify MAO-B/AChE multitarget hits and reduce the number of time- and resource-consuming in vitro and in vivo experimental tests.

#### 2.1.1. Protein Reliability Report

**Figure 2:**
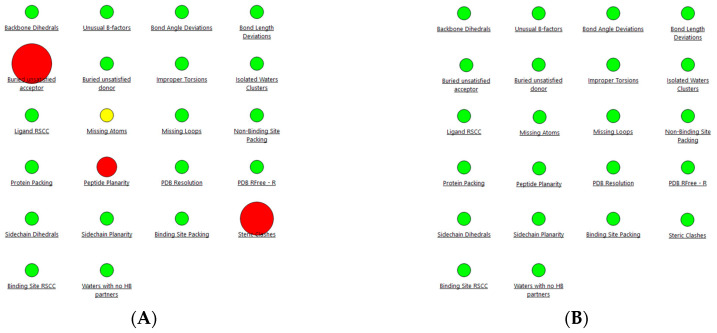

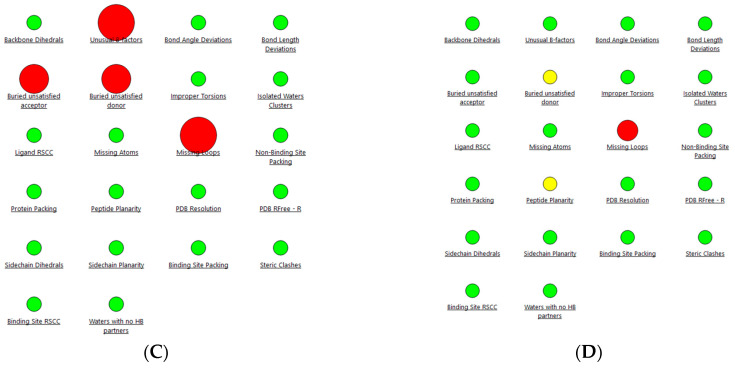
Protein reliability reports for MAO-B and AChE before and after structure preparation. Panels (**A**,**C**) show the initial protein reliability reports for MAO-B and AChE, respectively, highlighting structural deviations. Panels (**B**,**D**) display the corresponding results after applying the protein preparation workflow in Maestro. Regions marked in red with larger markers indicate deviations from acceptable structural parameters. The preparation notably improved structural reliability, as reflected by reduced deviations in panels (**B**,**D**).

Initially, the robustness of **2V5Z** and **4EY6** was evaluated using the Protein Reliability Report. As shown in Figure 2, the initially retrieved crystal structures of the enzymes MAO-B (PDB ID: **2V5Z**) and AChE (PDB ID: **4EY6**) showed several issues, including buried unsatisfied hydrogen-bond acceptors, missing loops, and steric clashes. Application of the protein preparation workflow (discussed in detail in the “Materials and Methods” Section) resolved these anomalies and resulted in cleaner, more reliable structures suitable for the following validation and docking studies.

The only remaining structural issues following the implementation of the missing loops in receptor **4EY6** were observed in the regions Pro258-Asn265 and Asp494-Pro498. These regions are located outside the active site and, therefore, will not be considered in the docking simulations. As a result, these deviations are not considered significant.

It was evident that refinement of the protein structures was essential, given the unacceptable structural values observed prior to preparation. Following protein preparation, we proceeded with redocking and ligand enrichment simulations.

#### 2.1.2. Ligand Enrichments Calculations

Ligand enrichment studies provide valuable insights into a protocol’s ability to distinguish active compounds from inactive ones. These studies often involve seeding known decoy molecules among active inhibitors and evaluating how well the docking method enriches true binders at the top of the ranked list [11]. We began with ligand enrichment analyses for the MAO-B and AChE enzymes by calculating AUC values. Afterward, we conducted a two-step consensus docking simulation: initial screening was performed using Glide, followed by re-docking with GOLD 5.3.

**Figure 3:**
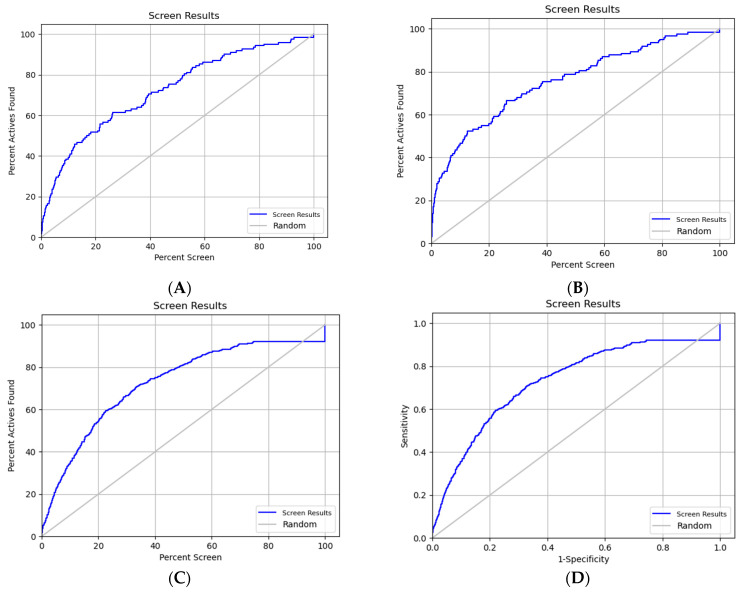
Enrichment plots illustrating the percentage of active compounds identified versus the percentage of screened compounds for MAO-B (panels (**A**,**B**)) and AChE (panels (**C**,**D**)). Panels (**A**,**C**) show results based on Glide XP docking scores, while panels (**B**,**D**) depict enrichment after MM/GBSA rescoring. The plots demonstrate the improved early recognition of active inhibitors following MM/GBSA rescoring, highlighting its effectiveness in prioritizing biologically relevant hits over decoys in virtual screening workflows.

After the preparation of the active inhibitors and decoy compounds, along with the previously prepared protein structures (as described in the preceding Section 2.1.1), the Glide docking scores were utilized to evaluate the protocol’s ability to distinguish active compounds from inactive ones or decoys. Analysis of the docking results yielded a receiver operating characteristic (ROC) curve with an area under the curve (AUC) value of 0.72 when the MAO-B enzyme was used, as shown in Figure 3A. This AUC value indicates that in 7.2 out of 10 cases, a randomly selected active compound received a higher docking score than a randomly selected inactive or decoy compound.

Subsequently, the entire dataset was rescored using the MM/GBSA function, which significantly improved the overall enrichment, particularly for MAO-B inhibitors. As illustrated in Figure 3B, the early enrichment factor for the tested dataset was enhanced. Following MM/GBSA rescoring, the revised ROC AUC value increased to 0.76.

**Figure 4:**
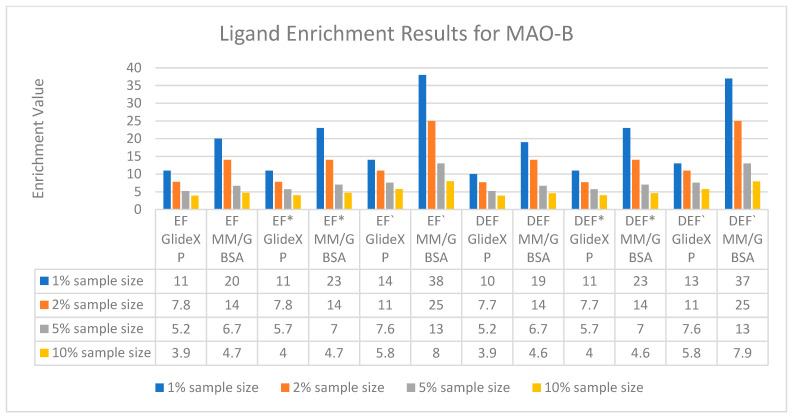
Enrichment factors and diversified enrichment values at different screened compound percentages for MAO-B. The plot compares enrichment metrics obtained using Glide XP docking scores versus MM/GBSA rescoring.

To further evaluate the improvement in early enrichment, a figure presenting various types of enrichment factors, including diversified enrichment factors, is provided in Figure 4.

The EF(1) and EF(2) values, calculated to be 11 and 7.8, respectively, when using GlideXP, indicate strong early recognition of active compounds over decoys in the virtual screening campaign. These values are especially meaningful in the context of structure-based drug discovery, as high enrichment factors within the top 1–2% of the dataset demonstrate the protocol’s ability to discriminate biologically relevant binders from non-binders early in the screening process. Such early enrichment is crucial in reducing the number of compounds requiring synthesis and experimental validation, thereby increasing the overall efficiency and cost-effectiveness of hit identification.

The ROC value obtained in this study further supports the robustness of the docking protocol and is comparable to that achieved by recent deep learning (DL)-based virtual screening methodologies [12]. Notably, while DL models have gained popularity for their learning capacity and performance across large datasets, our structure-based approach offers comparable or, in this case, superior early enrichment values. Specifically, the EF(1) and EF(2) values presented here are substantially higher than those reported in the aforementioned study, highlighting the optimized performance of our protocol in retrieving true actives within the top-ranked compounds.

To further evaluate the ranking efficiency, the modified enrichment factors (EF′s), which account for both diversity and positional accuracy of the actives, were also calculated. These values were found to be slightly higher than the corresponding standard EF values, suggesting a consistent top-ranking of multiple diverse active compounds within the dataset. Of particular note, EF′ for the top 1% of the ranked dataset reached a value of 38 after applying MM/GBSA rescoring—a significant improvement over the EF′ of 14 obtained using GlideXP alone. This demonstrates the added predictive value of post-docking rescoring methods in refining binding energy estimations and improving enrichment performance.

In addition to enrichment factors, hit rate analysis revealed that 40% of the top 50 ranked compounds were true actives, which represents a major improvement over typical hit rates in virtual screening campaigns. For context, recent studies report hit rates in the range of 11.4% with relatively small compound libraries and up to 22.4% when using expanded databases with enhanced chemical diversity [13]. The notably higher hit rate observed in our study confirms the practical applicability of our screening workflow and further emphasizes its potential to reduce resource-intensive downstream experimental assays. The ranking position of each active MAO-B inhibitor within the full screening list is provided in Supplementary Table S2, offering additional transparency and validation of compound prioritization.

**Figure 5:**
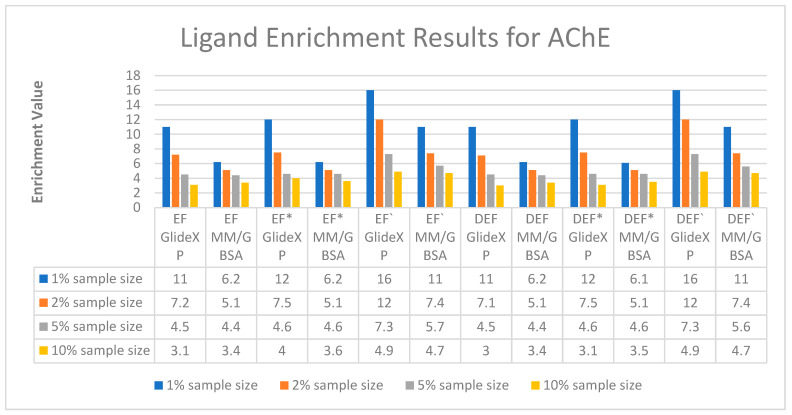
Enrichment factors and diversified enrichment values at different percentages of the screened compound dataset for AChE. The plot compares enrichment metrics obtained using Glide XP docking scores versus MM/GBSA rescoring.

The enrichment analysis for the AChE enzyme revealed a ROC value of 0.69 using GlideXP, as shown in Figure 3C. Notably, the enrichment performance improved following MM/GBSA rescoring, resulting in a slightly higher ROC value of 0.73 (Figure 3D) compared to 0.69 with GlideXP alone. This indicates that the application of MM/GBSA enhances the overall discriminative power of the virtual screening protocol for AChE inhibitors. A detailed comparison of various enrichment factors is provided in Figure 5. The exact ranks of each active AChE inhibitor are provided in Supplementary Table S3.

Overall, the results indicate that applying MM/GBSA rescoring after virtual screening with Glide XP leads to a considerable increase in the overall ROC values. Collectively, the findings underscore the effectiveness of the virtual screening protocol in achieving reliable early enrichment, accurate prioritization of active compounds, and enhanced hit rates, making it a valuable tool for accelerated discovery of both AChE and MAO-B inhibitors.

#### 2.1.3. Consensus Docking of the Top-Ranked Compounds

For consensus docking, we employed a two-step simulation: initial screening was performed using Glide—XP docking, followed by MM/GBSA rescoring of all ligands. Thereafter, the top-ranked compounds were docked with GOLD 5.3. The reliability of the predicted binding conformations was assessed by calculating the root mean square deviation (RMSD) values between the active binding poses obtained from both programs. We evaluated whether consensus docking could enhance the overall enrichment of the top-ranked compounds. Since MM/GBSA rescoring yielded better enrichment results compared to GlideXP scores, the top 1% of compounds ranked by MM/GBSA from the enrichment databases were subjected to further testing. The goal was to determine whether the consensus docking approach could more effectively differentiate true active compounds from false positives.

To do this, we compared the binding conformations of the compounds in the enzyme active sites as predicted by MM/GBSA and GOLD 5.3 and calculated the RMSD values between the two sets of poses. Consensus docking strategies commonly use RMSD thresholds as a criterion to assess pose similarity across different docking platforms. Based on the recent literature, we adopted the widely accepted threshold of 2.0 Å as our cutoff [14,15,16]. Compounds with RMSD values exceeding this threshold were considered to have inconsistent binding poses and were, thus, classified as false positives and excluded from further analysis.

After comparing the RMSD values between the docking poses generated by Glide and GOLD 5.3, it was found that 70 poses were accepted (with RMSD ≤ 2 Å), while 30 were rejected due to RMSD values exceeding 2 Å during the consensus docking simulations in MAO-B (Table 1). Notably, out of the 30 rejected poses, 25 were decoy molecules. In comparison, the consensus docking performed for AChE resulted in 72 rejected poses with RMSD values above 2 Å (Table 1). Among these, 64 were decoys, while only 8 were active compounds. Importantly, even for the rejected active poses in AChE, all the superimposed RMSD values remained below 3 Å—an observation further analyzed and discussed in the subsequent sections.

Relaxing the RMSD threshold from the standard 2 Å to 3 Å improves overall enrichment and leads to better results. Specifically, under the relaxed 3 Å threshold, all true active AChE inhibitors successfully passed the filter. However, this adjustment also allows more false positives (decoys) to be accepted—21 decoys were accepted compared to 43 rejected. This underscores the inherent trade-off between minimizing false positives and maximizing recovery of active compounds.

In our MAO-B enrichment analysis, increasing the RMSD cutoff to ≤3 Å retained two additional true inhibitors that were initially excluded by the stricter 2 Å criterion, highlighting the potential benefit of extending the RMSD threshold depending on the screening context.

Overall, employing a 3.0 Å RMSD cutoff in our consensus docking framework offers a balanced approach by retaining a greater number of true active compounds while still effectively reducing false positives. This threshold accommodates minor conformational variations during pose similarity assessment without significantly compromising screening reliability. Our results demonstrate that this relaxed RMSD cutoff enhances active compound recovery without a substantial increase in decoy acceptance, supporting its suitability for multi-software consensus docking workflows.

### 2.2. Virtual Screening of Pyrrole-Based Compounds

#### 2.2.1. Dataset Selection

The rationale for focusing on pyrrole-based compounds arises from our research group’s extensive experience in developing novel dual-acting inhibitors targeting both MAO-B and AChE, with the pyrrole scaffold as a central structural motif [17,18,19]. The recent literature further underscores the promise of pyrrole derivatives as highly potent and selective MAO-B inhibitors, highlighting their favorable interactions within the enzyme’s active site [17,18]. Additionally, published papers have reported new AChE inhibitors containing a pyrrole ring in their structure [20,21,22].

**Figure 6:**
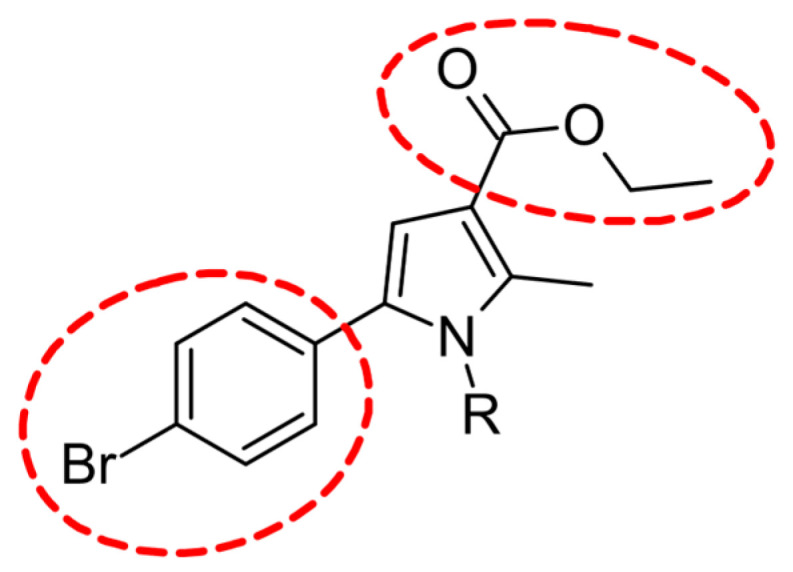
Previously identified steric clashes between ligand substituents and key residues within the binding pockets of MAO-B and AChE—highlighted by red circles.

To enhance the inhibitory activity of our pyrrole-based compounds against MAO-B and AChE, we methodically adjusted the substituents on the pyrrole scaffold. This involved trimming substituent sizes or introducing compact functional groups to better align with the spatial constraints of the enzymes’ active pockets. These modifications aimed to optimize binding interactions, thereby boosting both affinity and selectivity. Notably, our research group’s recent studies revealed that excessively bulky substituents on the pyrrole core reduced MAO-B inhibition, even when accounting for MAO-B’s larger active site compared to MAO-A [18,23]. This shows the importance of balancing substituent size with active site topology rather than relying solely on volume considerations. Therefore, the strategy was to remove the ethyl ester located at the third position and the 4-bromophenyl ring located at the fifth position in the pyrrole ring (Figure 6).

**Figure 7:**
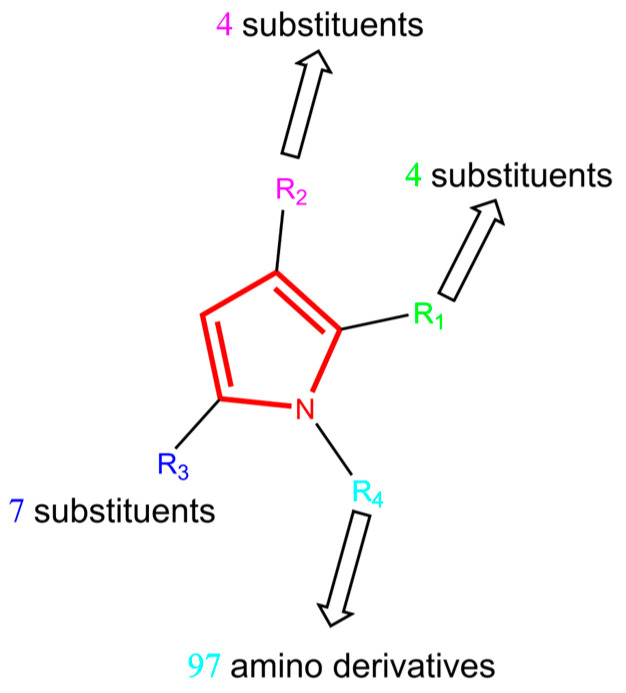
Dataset of 10,864 pyrrole-based compounds created for virtual screening.

The presented concept involves creating a small database of pyrrole-based compounds, which will be virtually screened using the consensus docking protocol optimized in this manuscript. The top-ranked candidates from this computational screening will then be synthesized and subjected to experimental evaluation for their inhibitory activity against MAO-B and AChE. In designing the new pyrrole-based compound library, substituents were introduced at the 1-, 2-, 3-, and 5-positions of the pyrrole ring, thereby generating a diverse set of structural analogs (Figure 7). This approach aims to explore the impact of various substitution patterns on enzyme binding and inhibitory potency, ultimately guiding the identification of promising lead compounds for further development.

The specific fragments used to construct the dataset are detailed in Supplementary Table S1. A total of 10,864 pyrrole-based molecules were generated. During fragment selection, synthetic feasibility was evaluated to ensure experimental accessibility. The synthetic accessibility of the pyrrole-based compounds was calculated using SwissADME, which provides a “synthetic accessibility score” ranging from 1 to 10, where 1 indicates compounds that are very easy to synthesize and 10 indicates those that are very difficult. Our dataset demonstrated synthetic accessibility scores ranging from 1.49 to 5.01, indicating that the majority of the generated molecules in our library are predicted to be easy to moderately challenging for organic synthesis. This suggests a favorable balance between molecular complexity and synthetic tractability, supporting the practical feasibility of synthesizing the top-ranked compounds. The three-dimensional structures of the ligands were computationally generated and prepared for molecular docking following the protocols outlined in the “Materials and Methods” Section.

#### 2.2.2. Virtual Screening

**Figure 8:**
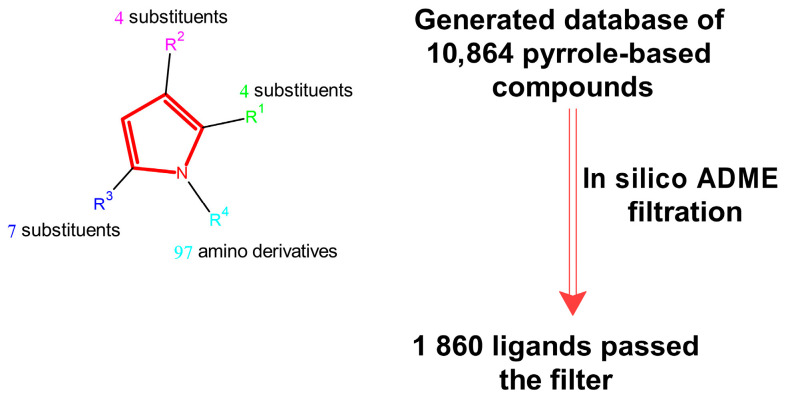
Initial in silico ADME filtration of the pyrrole database.

The initial ligand filtering was based on ADME properties, applying the following criteria: Rule of Five violations less than one (RuleOfFive < 1), polar surface area below 140 Å^2^ (PSA < 140), and predicted blood–brain barrier partition coefficient in the range −3.0 to 1.2 (QPlogBB −3.0 to 1.2). These thresholds are widely recognized for identifying compounds with favorable drug-like and pharmacokinetic profiles prior to virtual screening [24]. A PSA below 140 Å^2^ is considered optimal for membrane permeability, while stricter limits (<90 Å^2^) are preferred for CNS penetration. The selected QPlogBB interval reflects the range for 95% of marketed drugs, ensuring adequate brain access without excessive exposure and minimizing risks of poor permeability or off-target CNS effects. Most of the pyrrole-based ligands were filtered out due to one or more violations of Lipinski’s Rule of Five; therefore, only 1860 molecules remained for further investigation (Figure 8).

The compounds that passed the initial ADME filter were subjected to virtual screening using the optimized consensus docking protocol involving the licensed software Glide and GOLD 5.3. The dataset was first screened with the Glide XP docking option, after which all the poses were re-evaluated using MM/GBSA. Subsequently, a second software, GOLD 5.3, was applied to validate the hit structures. In this study, we used an RMSD threshold of 3 Å, as it produced the best results during validation of the consensus docking protocols (Table 2). The top 20 structures were selected based on the best MM/GBSA scores, while an additional 7 ligands were retained due to lower docking scores but favorable interactions within the receptor’s active site (Supplementary Figure S1).

Within MAO-B, the compounds exhibited MM-GBSA scores ranging from approximately −50 to −62 kcal/mol, surpassing the reference inhibitor selegiline, which showed a MM-GBSA of −55.28 kcal/mol. This suggests several hits possess comparable or stronger binding affinities to MAO-B than selegiline, which will be further validated by in vitro studies. In the AChE docking results, the top MM-GBSA scores were notably stronger than those observed for the hits in MAO-B, ranging from about −47 to −58 kcal/mol. However, the clinically approved inhibitors, donepezil and galanthamine, displayed higher scores of −83.76 kcal/mol and −62.58 kcal/mol, respectively, in AChE, confirming the docking system’s effective discrimination of high-affinity ligands. Notably, several new compounds, such as **EM-DC-13** and **EM-DC-14**, showed MM-GBSA values close to or exceeding −58 kcal/mol, suggesting potent AChE inhibition. Compounds with RMSD values below 3 Å, when comparing their docked conformations to the reference active conformations, were selected for further evaluation.

**Figure 9:**
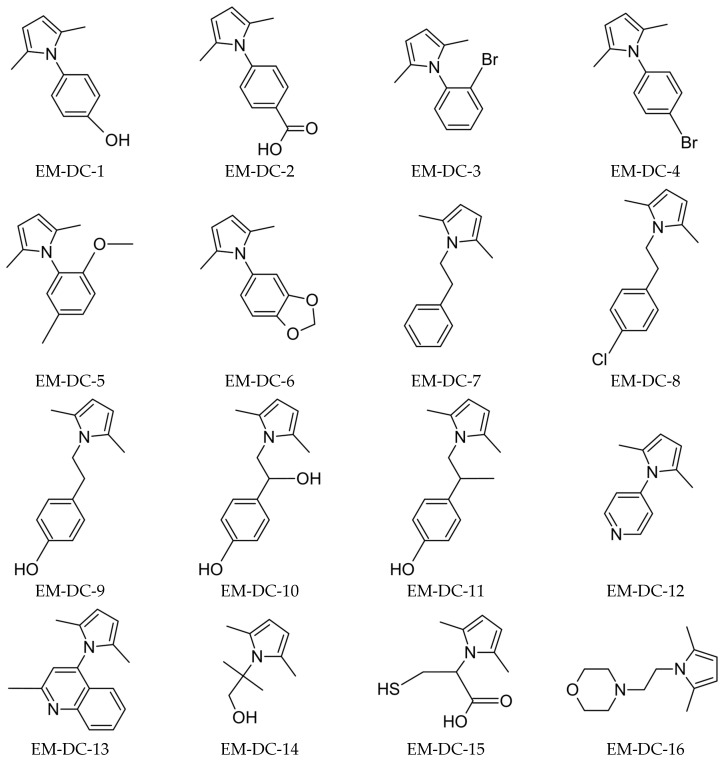

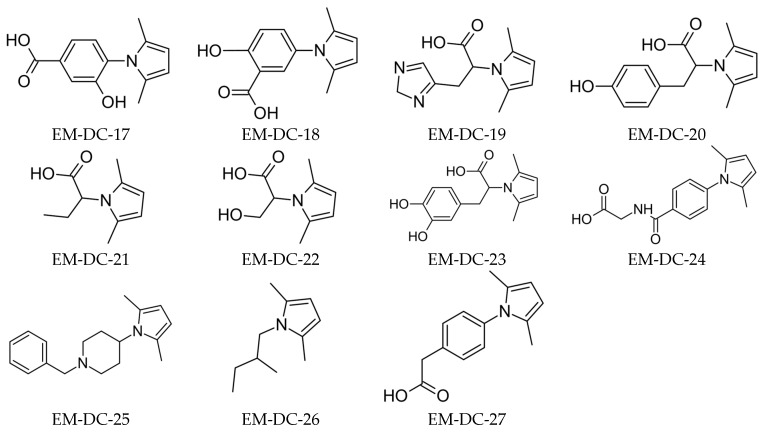
Two-dimensional structures of the top-ranked dual-acting MAO-B/AChE inhibitors identified by consensus docking.

The 2D structures of the identified hits are shown in Figure 9.

The in silico calculations hypothesized that the 2,5-dimethylpyrrole ring substituted at the first position with various fragments is well suited to bind within the active site of MAO-B. Several different moieties were identified as potentially active dual MAO-B/AChE inhibitors, including morpholine, 2-methylquinoline, phenylethylamine, and multiple free carboxylic acid groups. The presence of carboxylic acid moieties may contribute to stronger binding through the formation of hydrogen bonds with key active-site amino acid residues. This capability enhances ligand stabilization within the enzyme active site, likely improving inhibitory potency. These findings align with previous studies highlighting the importance of hydrophobic and polar interactions, including hydrogen bonding and π-π stacking, in the enzyme’s substrate recognition and inhibitor selectivity [25].

The SA scores of the top-ranked compounds ranged from 1.49 to 2.94, indicating that these molecules are theoretically easy to synthesize. This range reflects a favorable synthetic profile, with low complexity and common molecular fragments that are frequently encountered in commercially available compounds. Such scores suggest that the hit compounds from our pyrrole dataset possess practical synthetic feasibility, making them suitable candidates for further chemical synthesis and biological evaluation.

### 2.3. Synthesis of the Leader Structures

**Scheme 1:**
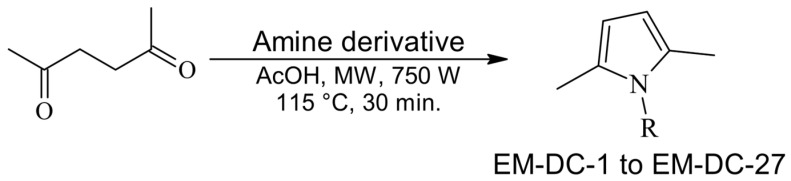
Synthetic route for the preparation of the identified hit pyrrole-based compounds.

For the synthesis of the identified pyrrole-based hit compounds, we employed the widely used Paal–Knorr condensation reaction. This method involves the cyclization of amine derivatives with a 1,4-diketone fragment to form the pyrrole ring. The Paal–Knorr condensation reaction is favored for its simplicity, mild conditions, and high yields, making it an effective strategy for synthesizing diverse pyrrole derivatives [26]. The general reaction scheme is depicted in Scheme 1.

The synthesized compounds have been previously reported by numerous research groups [27,28,29,30,31,32,33,34,35]; therefore, we identified the compounds by testing their IR, ^1^H-NMR, and LC/MS spectra. The obtained spectral data corresponded well with the reported values, confirming the structural integrity of the synthesized molecules (Supplementary Figure S2).

Notably, the reactions were performed using a professional microwave reactor, which strictly controls reaction time and temperature. This reactor enabled significantly higher yields and substantially faster reaction times compared to conventional heating methods, highlighting the efficiency and reproducibility of microwave-assisted synthesis. Microwave (MW) irradiation significantly accelerates chemical reactions by providing rapid and uniform heating directly to the reaction mixture. Compared to conventional heating, microwaves reduce reaction times from hours to minutes and often improve product yields and purity [36].

### 2.4. In Vitro Enzymatic Activity Evaluation of AChE, BChE, MAO-A, and MAO-B

To validate the obtained in silico data, we conducted in vitro studies employing the main enzymes connected to the development and progression of PD—MAO-A, MAO-B, AChE, and BChE. These enzymes play pivotal roles in neurodegenerative pathways, including oxidative stress mediation, dopamine metabolism regulation, and cholinergic system dysfunction. Detailed descriptions of the experimental setup, including sample preparation, assay parameters, and concentrations, are provided in the “Materials and Methods” Section. The results of these enzymatic inhibition studies are presented in Table 3 and further analyzed in the subsequent discussion.

The obtained in vitro results generally correlate with the in silico calculations, which lends support to the reliability of our virtual screening approach. Out of the 27 hits identified through docking, 7 compounds were confirmed as active AChE inhibitors, resulting in a hit rate of 25.93%. The threshold we used to classify a ligand as a hit was an IC_50_ value of less than 100 µM. An exception was made for the compound **EM-DC-12**, which exhibited an IC_50_ value of 102.57 ± 5.85 µM, and it was calculated as a hit.

For MAO-B inhibitors, 12 out of 27 compounds were found to be active, corresponding to a hit rate of 44.44%. When compared to recent studies on virtual screening, our hit rates were above the expected range reported for prospective docking campaigns. Typical hit rates in prospective virtual screening studies vary widely, often ranging from 1% to 40%, with higher rates sometimes observed when using advanced docking protocols combined with machine learning or free energy calculations [37].

A notable discrepancy was observed in some hit compounds that exhibited MM-GBSA binding free energy scores comparable to or exceeding that of selegiline but showed significantly weaker AChE inhibition compared to galantamine. This disparity likely reflects inherent differences in the binding site characteristics and interaction requirements of MAO-B versus AChE enzymes.

Among the 27 compounds identified as top hits by virtual screening against MAO-B, several demonstrated promising inhibitory activities with varying selectivity profiles toward the target. Compounds **EM-DC-2** and **EM-DC-13** notably exhibited potent inhibition of AChE, with inhibition percentages at 200 µM of 93.60 ± 4.66% and 91.15 ± 1.22%, respectively, corresponding to IC_50_ values of 0.75 ± 0.06 µM and 3.81 ± 0.26 µM. These values indicate a strong affinity and inhibitory capacity of these compounds toward AChE. In contrast, their BChE inhibitory activity tends to be moderate to low with IC_50_ above 10 µM, implying selectivity toward AChE over BChE. This selectivity is desirable in targeting cholinergic dysfunction associated with neurodegenerative disorders while potentially minimizing side effects linked to BChE inhibition [38].

Regarding MAO inhibition, several compounds showed considerable activity against MAO-B, consistent with the virtual screening results focusing on this enzyme. For instance, **EM-DC-2** displayed an IC_50_ of 0.444 ± 0.10 µM for MAO-B, accompanied by strong inhibition at 1 µM concentration (75 ± 7.1%). Similarly, **EM-DC-19** also showed significant MAO-B inhibition with an IC_50_ of 0.299 ± 0.10 µM and 50 ± 7.1% inhibition at 1 µM. Several other compounds, such as **EM-DC-12** and **EM-DC-7,** exhibited MAO-B IC_50_ values in the submicromolar range, reinforcing their potential as selective MAO-B inhibitors. These activities align well with the aim of identifying novel inhibitors for Parkinson’s disease, given MAO-B’s critical role in dopamine metabolism and neurodegeneration.

The MAO-A inhibitory activity across most compounds was comparatively less potent, with many showing IC_50_ values greater than 100 µM, indicating lower affinity or selectivity for MAO-A. This selectivity might contribute to reduced undesired side effects related to MAO-A inhibition, such as hypertensive crises caused by dietary tyramine interaction.

Compounds **EM-DC-19** and **EM-DC-27** demonstrated selective inhibitory activity toward MAO-B with IC_50_ values of 0.299 ± 0.10 µM and 0.344 ± 0.10 µM, respectively, while exhibiting moderate to low inhibition of AChE and BChE enzymes. This selectivity profile highlights their potential as targeted MAO-B inhibitors, which is valuable given MAO-B’s key role in the pathogenesis of neurodegenerative diseases such as Parkinson’s disease. Additionally, the moderate cholinesterase inhibition by these compounds suggests a multitarget therapeutic potential, addressing both monoamine oxidase-mediated dopamine metabolism and cholinergic dysfunction, two critical mechanisms in neurodegeneration [39].

These promising dual activities position **EM-DC-19** and **EM-DC-27** as candidates for further investigation. Accordingly, they were subjected to extended evaluation in rat brain synaptosomes and mitochondria to explore their functional effects in more complex biological systems and to assess their neuroprotective capacity under physiologically relevant conditions.

### 2.5. SAR Analysis

The presence of a hydroxyl (-OH) group in the amine substituent, observed in compounds EM-DC-1, EM-DC-9, EM-DC-10, EM-DC-17, EM-DC-18, EM-DC-20, and EM-DC-23, generally resulted in low to moderate inhibitory activity against AChE. This suggests that while the -OH moiety may contribute to hydrogen bonding interactions, its effect alone is insufficient to significantly enhance AChE binding affinity or enzyme inhibition [40]. However, the -OH group appeared to contribute beneficially to MAO-B inhibition but only in the specific case of EM-DC-20, where it was accompanied by a carboxylic acid (-COOH) group. The coexistence of these two functional groups may create a synergistic effect by promoting stronger or more stable interactions within the MAO-B active site, such as through additional hydrogen bonding or ionic interactions with key amino acid residues or the flavin adenine dinucleotide (FAD) cofactor.

The presence of halogen atoms in the studied compounds did not result in enhanced AChE inhibition, indicating that halogenation alone may not significantly improve binding affinity or enzyme blockade within this series. Among these, only EM-DC-3 demonstrated moderate MAO-B inhibitory activity, suggesting that specific halogen substitutions, depending on their position and nature, may contribute selectively to MAO-B inhibition rather than broad-spectrum activity [41].

The significant difference in AChE inhibitory potency between **EM-DC-2** and **EM-DC-19**, despite both containing the carboxylic acid group, can be explained by their structural and functional group contexts. **EM-DC-2** features a carboxylic acid directly attached to an aromatic ring, enabling optimal positioning for strong hydrogen bonding and ionic interactions with catalytic residues in the AChE active site. This direct aromatic conjugation also facilitates crucial π-π stacking interactions with key residues like Trp86, stabilizing the inhibitor binding and enhancing potency. Conversely, **EM-DC-19** has the carboxylic acid attached via a flexible propanoic acid side chain combined with an imidazole substituent. This flexible linkage likely reduces effective positioning and stable hydrogen bond formation within the enzyme pocket, while the imidazole group may introduce steric hindrance or disrupt favorable aromatic interactions. Together, these factors weaken binding affinity and lead to an approximately 100-fold lower inhibition potency compared to **EM-DC-2**. The analysis highlights that not just the presence of a functional group like -COOH, but its spatial arrangement, rigidity of the scaffold, and complementary substituent effects critically determine AChE inhibitory activity. **EM-DC-2′**s planar structure and direct aromatic carboxyl substitution create a more optimal binding conformation, resulting in substantially enhanced potency.

Notably, compounds containing an unsubstituted carboxylic acid group, namely **EM-DC-2**, **EM-DC-17**, **EM-DC-18**, **EM-DC-19**, **EM-DC-21**, **EM-DC-22**, **EM-DC-23**, **EM-DC-24**, and **EM-DC-27**, consistently exhibited enhanced inhibitory effects against both AChE and MAO-B. This emphasizes the critical role of the -COOH functionality in improving the overall binding affinity and enzymatic blockade. The enhanced activity may arise from the ability of the carboxylic acid to form strong hydrogen bonds and ionic interactions with residues within the active cavities of both enzymes, thereby stabilizing the ligand–enzyme complex [42]. Among these, EM-DC-2 and EM-DC-19 were identified as the most potent dual inhibitors, demonstrating superior efficacy in modulating both cholinergic and monoaminergic pathways. These results highlight that including carboxylic acid groups in the molecules is an important factor for creating drugs that can target multiple enzymes involved in neurodegenerative diseases. Overall, the most prominent selective MAO-B inhibitors with good to moderate AChE inhibitory effects contained a -COOH moiety (**EM-DC-19** and **EM-DC-27**). These compounds provide a promising scaffold for further structural modifications to enhance their multitarget activity in future studies.

### 2.6. QSAR Study of BChE Inhibition

QSAR modeling was conducted exclusively for BChE, as sufficient experimental IC_50_ data were available only for this enzyme. Using the GA-MLR method, several QSAR models were generated with different descriptors. The best model was selected based on its statistical parameters, showing good robustness and reliability. The final model includes three descriptors: SHBint2, ETA_Epsilon_5, and nAtomP (Table 4).

The model showed good performance with R^2^ = 0.867, R^2^adj = 0.830, Q^2^ = 0.735, and R^2^test = 0.600. Variance Inflation Factor (VIF) values were all below 5 (1.004–1.840), indicating no multicollinearity among descriptors. T-test values (|3.94|–|6.87|) confirmed that each descriptor significantly contributes to the model.

Each descriptor affects BChE inhibition differently:

1. SHBint2, a surface-based hydrogen bond descriptor, has a positive coefficient (0.197), suggesting that increased hydrogen bonding enhances inhibitory activity.
2. ETA_Epsilon_5 has a negative coefficient (−16.205), indicating that higher electronic spatial features decrease activity.
3. nAtomP, representing the number of atoms in the largest π-system, has a positive coefficient (0.181), showing that extended π-conjugation favors stronger interactions with BChE.

The model equation is as follows:

pIC50 = 14.588 + 0.197 **SHBint2** − 16.205 **ETA_Epsilon_5** + 0.181 **nAtomP**

The Y-randomization test confirmed that the model is not due to chance. None of the 200 randomized trials achieved comparable R^2^ or Q^2^ values (mean R^2^ = 0.208, Q^2^ = 0.542), supporting the model’s predictive validity.

**Figure 10:**
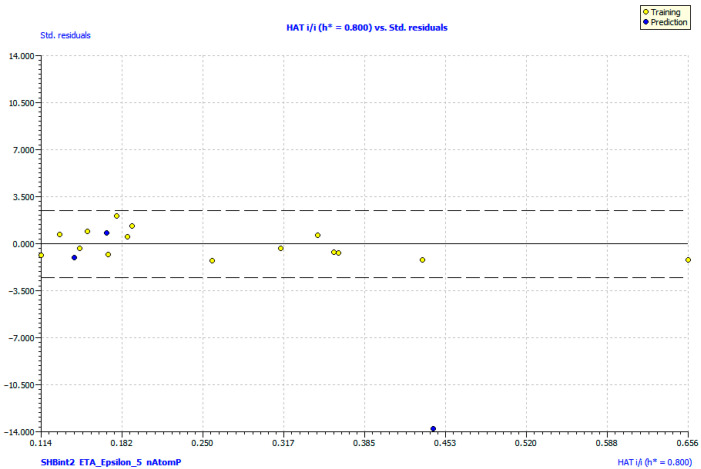
William’s plot of leverage-normalized residuals for the developed model.

The applicability domain (AD) was evaluated using a Williams plot. All the compounds from both the training and test sets fell within the domain, with leverage values below the critical threshold (h* = 0.800) and standardized residuals within ±2.45 (Figure 10). This confirms that the model predictions are reliable and meaningful for the studied chemical space.

### 2.7. Molecular Docking in MAO-B and AChE

To gain deeper insight into the intermolecular interactions underlying the potency of the dual-acting MAO-B/AChE inhibitors EM-DC-19 and EM-DC-27, we performed a detailed analysis of the bonds formed between the ligands and the enzymes’ active sites. The key interactions are illustrated in both 2D and 3D panels provided in Supplementary Figure S3. In the active site of MAO-B, most stabilizing interactions were hydrophobic in nature, consistent with the enzyme’s largely hydrophobic substrate-binding cavity [25]. Both compounds prominently interacted with Tyr398 and Tyr435, which together form the aromatic cage critical for substrate recognition and ligand stabilization. Additionally, the pyrrole-based ligands exhibited favorable interactions within the substrate cavity, engaging amino acid residues Cys172, Ile198, and Ile199. Importantly, EM-DC-19 formed a strong hydrogen bond with Cys172 at a distance of 2.54 Å, enhancing binding affinity. In contrast, the benzene moiety of EM-DC-27 established a significant π-π stacking interaction with Tyr326, an amino acid known to play a pivotal role in stabilizing MAO-B/ligand complexes [43]. Both ligands also formed polar contacts with Gln206, further contributing to the ligands’ binding stabilization in the active site.

In the active site of AChE, the primary interactions of the two ligands occurred with the highly conserved residue Trp86, a key component of the enzyme’s aromatic gorge critical for ligand recognition and stabilization [44]. **EM-DC-19** engaged Trp86 through π-π stacking interactions specifically involving its pyrrole fragment, forming strong aromatic contacts. **EM-DC-27** exhibited an even more solid interaction, forming two π-π stacking contacts with Trp86 via both its pyrrole ring and benzene moiety, thereby enhancing its binding affinity. Beyond these hydrophobic interactions, **EM-DC-19** established multiple hydrogen bonds that further stabilized the ligand within the active site. Notably, it formed a direct hydrogen bond with Glu202 and two water-mediated hydrogen bonds involving Gly122 and Ser203. These interactions were predominantly associated with the carboxylic moiety of **EM-DC-19**, underscoring the critical role of this functional group in mediating stable binding to AChE through hydrogen bonding networks. In contrast, **EM-DC-27** (MM/GBSA score −43.81 kcal/mol) lacks these additional hydrogen-bonding interactions, which helps explain the observed difference in the experimental inhibitory potency, with **EM-DC-19** (MM/GBSA score -55.43 kcal/mol) displaying a significantly lower IC_50_ value of 76.15 ± 6.12 µM compared to **EM-DC-27′s** IC_50_ of 375.20 ± 52.99 µM. This variation in binding affinity is also reflected in their docking scores, where **EM-DC-19** scored more favorably.

### 2.8. Effects of the Compounds EM-DC-19 and EM-DC-27 on Isolated Rat Brain Synaptosomes

**Figure 11:**
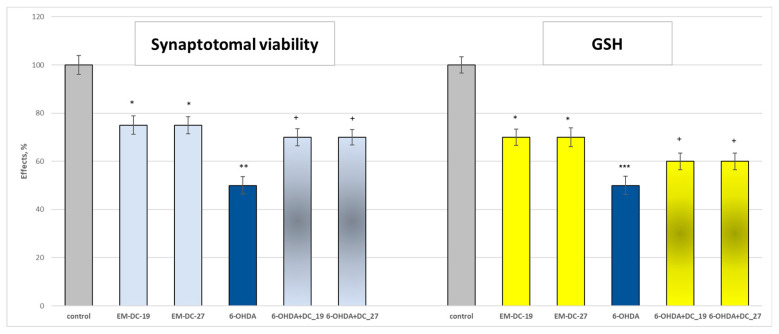
Effects of the compounds **EM-DC-19** and **EM-DC-27** (at a concentration of 100 μM), administered alone and in combination (at a concentration of 50 μM) with 6-OHDA (150 μM), on synaptosomal viability and GSH level. * *p* < 0.05; ** *p* < 0.01; *** *p* < 0.001 vs. control (non-treated synaptosomes) + *p* < 0.05 vs. control (6-OHDA) (mean values from triplicate experiments).

After identifying the two selective MAO-B inhibitors with additional AChE inhibiting effects, we followed with studies on isolated rat brain synaptosomes and microsomes. In isolated rat brain synaptosomes, administered alone at a concentration of 100 μM, the compounds **EM-DC-19** and **EM-DC-27** revealed low statistically significant neurotoxic effects on synaptosomal viability and level of reduced glutathione compared to the control (non-treated synaptosomes) (Figure 11). Compounds **EM-DC-19** and **EM-DC-27** decreased synaptosomal viability by 25% and GSH level by 30% compared to the control.

Because 6-OHDA has high affinity for the dopamine transporter, it selectively damages dopaminergic neurons by generating reactive oxygen species (ROS). Often, 6-OHDA is administered unilaterally because the lethality of bilateral injections of this compound into the striatum is higher. On the other hand, under the action of MAO-B, 6-OHDA is metabolized to the reactive metabolite p-quinone, which also generates an overproduction of ROS on the one hand, and on the other, it reduces to a significant extent the level of GSH, the main cellular protector [45].

Administered alone, 6-OHDA (at a concentration of 150 μM) revealed a statistically significant neurotoxic effect by decreasing the synaptosomal viability and GSH level by 50% compared to the control (non-treated synaptosomes) (Figure 11).

In this model of neurotoxicity, both compounds **EM-DC-19** and **EM-DC-27** (at a concentration of 50 µM) revealed good statistically significant neuroprotective effects. Both of them preserved synaptosomal viability and GSH level, respectively, with 40% and 20% compared to the control (toxic 6-OHDA).

### 2.9. Effects of the Compounds EM-DC-19 and EM-DC-27 on Isolated Rat Brain Mitochondria

**Figure 12:**
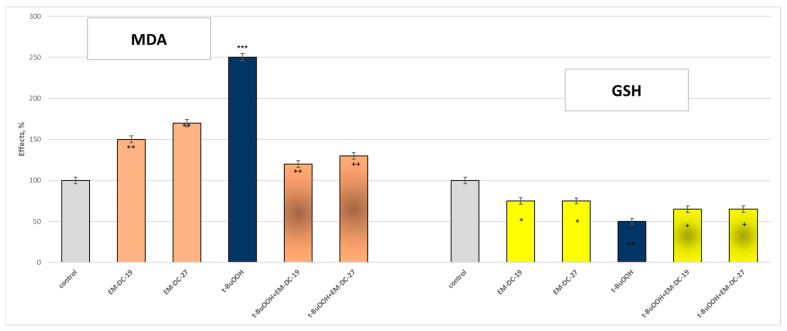
Effects of the compounds **EM-DC-19** and **EM-DC-27** (at a concentration of 100 μM), administered alone and in combination (at a concentration of 50 μM) with t-BuOOH (75 μM), on MDA production and GSH level in isolated rat brain mitochondria. * *p* < 0.05; ** *p* < 0.01; *** *p* < 0.001 vs. control (non-treated mitochondria) + *p* < 0.05; ++ *p* < 0.01 vs. control (t-BuOOH) (mean values from triplicate experiments).

In isolated rat brain mitochondria, administered alone at a concentration of 100 μM, the compounds **EM-DC-19** and **EM-DC-27** revealed low statistically significant neurotoxic effects on MDA production and level of reduced glutathione compared to the control (non-treated mitochondria) (Figure 12). Compounds **EM-DC-19** and **EM-DC-27** decreased GSH level by 25% compared to the control. **EM-DC-19** increased MDA production by 50% and **EM-DC-27** by 70% compared to the control.

A suitable model of oxidative stress is the use of *tert*-butyl hydroperoxide (*t*-BuOOH), which has mitochondrial and microsomal metabolism. Its metabolism to free radical formation proceeds through several steps. In microsomal suspension, in the absence of NADPH, it undergoes a one-electron oxidation to a peroxyl radical (reaction 1), whereas in the presence of NADPH, it undergoes a one-electron reduction to an alkoxyl radical (reaction 2). In isolated mitochondria and in intact cells, *t*-BuOOH undergoes β-cleavage to a methyl radical (reaction 3).

All these radicals trigger a process of lipid peroxidation and reduce the level of reduced glutathione [46].

(CH_3_)_3_COOH → (CH_3_)_3_COO- + e- + H+ (reaction 1)

(CH_3_)_3_COOH + e- → (CH_3_)_3_CO- + OH- (reaction 2)

(CH_3_)_3_CO- → (CH_3_)_2_CO + -CH_3_ (reaction 3)

Administered alone, *t*-BuOOH (at a concentration of 75 μM) revealed a statistically significant neurotoxic effect by decreasing the GSH level by 50% and increasing MDA production by 150% compared to the control (non-treated mitochondria) (Figure 12).

In this model of neurotoxicity, both compounds **EM-DC-19** and **EM-DC-27** (at a concentration of 50 µM) revealed good statistically significant neuroprotective and antioxidant effects. Both of them preserved GSH level by 30% compared to the control (toxic t-BuOOH). **EM-DC-19** decreased MDA production by 52% and **EM-DC-27** by 48% compared to the toxic agent.

### 2.10. Effects of the Compounds EM-DC-19 and EM-DC-27 on Isolated Rat Brain Microsomes

**Figure 13:**
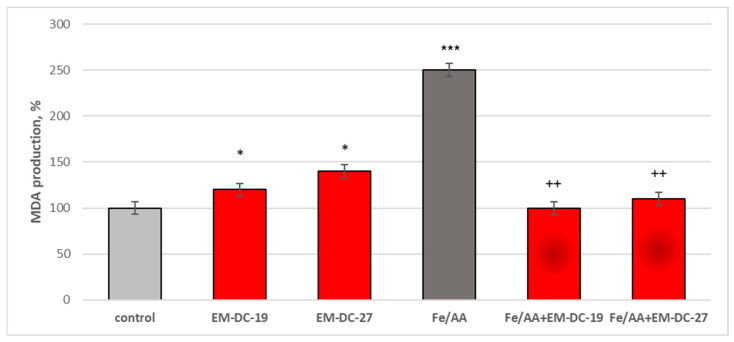
Effects of the compounds **EM-DC-19** and **EM-DC-27** (at a concentration of 100 μM), administered alone and in combination (at a concentration of 50 μM) with Fe/AA, on MDA production in isolated rat brain microsomes. * *p* < 0.05; *** *p* < 0.001 vs. control (non-treated microsomes); ++ *p* < 0.01 vs. control (Fe/AA) (mean values from triplicate experiments).

In isolated rat brain microsomes, administered alone at a concentration of 100 μM, the compounds **EM-DC-19** and **EM-DC-27** revealed again low statistically significant pro-oxidant effects on levels of malondialdehyde (MDA) compared to the control (non-treated microsomes) (Figure 13). Compound **EM-DC-19** increased MDA production by 20% and **EM-DC-27** by 40% compared to the control.

A classic model of lipid peroxidation is iron/ascorbate-induced peroxidation. Administered alone, the combination of iron with ascorbic acid increased MDA production by 139% compared to the control. In this combination, a Fenton reaction occurred, which led to increased production of OH• radicals.

In this model of rat brain microsomes, which is a model of lipid membrane, both compounds **EM-DC-19** and **EM-DC-27** revealed good statistically significant antioxidant effects. **EM-DC-19** decreased MDA production by 60% and **EM-DC-27** by 56% compared to the toxic agent (Figure 13).

The data revealed that the dual-acting compounds **EM-DC-19** and **EM-DC-27** exhibited significant neuroprotective and antioxidant effects. Overall, these compounds provide a promising scaffold for further structural modifications, due in part to the presence of a free -COOH moiety. This functional group offers opportunities for chemical adjustment to enhance their multitarget activity in future studies aimed at developing more effective neuroprotective agents.

### 2.11. In Silico ADME and Toxicological Profile of the Leader Compounds

To further characterize the pharmacokinetic and toxicological profiles of the lead compounds, in silico studies were performed using Derek Nexus for toxicity prediction and QikProp for ADME properties (Table 5).

The ADME profiles indicate that both compounds possess favorable properties for CNS penetration, with polar surface areas consistent with known thresholds for brain accessibility. However, the presence of carboxylic acid groups in **EM-DC-19** and **EM-DC-27** imparts increased polarity, which may limit their BBB permeability. The predicted QPlogBB values (−0.624 for **EM-DC-19** and −0.451 for **EM-DC-27**) suggest moderate CNS penetration, with **EM-DC-27** being relatively more lipophilic and likely to traverse biological membranes more readily.

Neither compound violated Lipinski’s rule of five, supportive of good oral bioavailability potential. However, **EM-DC-27** triggered a hepatotoxicity alert linked to its 2-arylacetic acid moiety, a structural feature associated with the hepatotoxic effects observed in some nonsteroidal anti-inflammatory drugs (NSAIDs) of this class. Given this, further chemical optimization of **EM-DC-27** or strategic modification of the carboxyl group (e.g., prodrug formation and ester derivatives) may be necessary to mitigate potential toxicity while enhancing CNS delivery.

In contrast, EM-DC-19 displayed a cleaner toxicological profile with no predicted toxicity alerts, as none of the assessed endpoints triggered any alerts (Supplementary Table S6). Additional theoretical mutagenicity evaluation using Derek Nexus predicted the compound to be inactive in bacterial mutagenicity tests. Derek Nexus is a well-validated expert-knowledge-based in silico tool that provides reliable qualitative predictions for mutagenicity endpoints, aligning with regulatory frameworks for early toxicity assessment. This in silico results supports the favorable safety profile of **EM-DC-19** and its suitability for further development.

## 3. Materials and Methods

### 3.1. Virtual Screening

The crystal structures of MAO-B and AChE (PDB codes **2V5Z** and **4EY6**) were retrieved from the Protein Data Bank (PDB). Protein preparation was carried out using the protein preparation module in Maestro, which added hydrogen atoms, corrected missing loops, and minimized the protein structures. The Protein Reliability Report module, integrated within the Maestro software suite (Schrödinger, NY, USA), was employed to compare the unprepared and prepared proteins. This module serves as a robust tool for evaluating the structural integrity and reliability of crystal structures used in computational research. It identifies potential structural issues such as steric clashes, missing loops, poorly resolved regions, and geometric deviations, all of which can compromise the accuracy of subsequent virtual screening studies. Overall, the module provided essential insights into the quality and reliability of the selected X-ray structures.

The docking programs Glide (Schrödinger) and GOLD (Genetic Optimization for Ligand Docking) were employed in this study. The scoring function applied in GOLD 5.3 was ChemPLP, while XPscore was used for Glide. Molecular Mechanics/Generalized Born Surface Area (MM-GBSA) recalculations with Prime were performed to assess the free binding energies of the obtained complexes. The calculations were carried out using the OPLS3 force field in combination with the VSGB solvation model. The grid box was generated around the co-crystallized ligands in the X-ray proteins.

For ligand enrichment, active structures were used, while decoys were obtained from the Database of Useful Decoys: Enhanced (DUD-E). The decoys possess physical properties similar to the active compounds but are inactive. For MAO-B, 169 actives and 6931 decoys were downloaded, whereas for AChE, 664 actives and 26,373 decoys were retrieved.

We performed calculations of various enrichment factors (EFs) to quantify the reliability and performance of our docking program in virtual screening. Specifically, we calculated the classical enrichment factor (EF); the modified enrichment factor (EF’); and the Enhanced Enrichment Factor (EF*), Difference Enrichment Factor (DEF), Enhanced Difference Enrichment Factor (DEF*), modified Difference Enrichment Factor (DEF’), and receiver operating characteristic (ROC) curve analysis.

The **EF** measures the fold increase in active compounds found in a subset of the screened database relative to a random distribution. It does not take into account the rank positions of the active compounds. The **EF’** emphasizes early recognition by giving higher values when active compounds appear near the top of the ranked list.

The formulas for both are as follows:

EF = (Hits_sampled_/Hits_total_)/(N_sampled_/N_total_),

where Hits_sampled_ are the actives located in the chosen dataset (N_sampled_). N_total_ corresponds to all compounds included in the dataset, while Hits_total_ is the number of active molecules seeded in the decoys.

EF`(N) = (50%/APR_sampled_) × (Hits_sampeld/_Hits_total_),

where N is the percent of the active compounds; ARP stands for “average percentile rank” of Hits_total_. In this study we looked at several seedings of the active compounds.

The EF* is adjusted to consider the distribution of active compounds across different ranked ranges to capture enrichment over a broader selection window. DEF compares the enrichment of active compounds in a specific sample relative to a control or baseline enrichment. DEF* and DEF’ are variations in DEF incorporating rank weights (similar to EF*) and early recognition emphasis (similar to EF’).

The fragments, used in the generation of the small database of 10,000+ pyrrole-based structures, were created by using the R-group creator in Maestro. The fragments were manually drawn based on the possibility of a chemical reaction occurring. The utilized fragments are provided in Supplementary Table S1. The chemical structures were converted to the corresponding three-dimensional (3D) structure with the LigPrep module (Schrödinger Release 2025-1: LigPrep, Schrödinger, LLC, New York, NY, USA, 2025). Utilizing LigPrep, hydrogen bonds, tautomers, and ionization states at pH 7.0 ± 0.2 were generated. Furthermore, the energies of the ligands were minimized by applying the OPLS4 force field. The ADME filtering was conducted with the QuikProp in Maestro.

The synthetic accessibility (SA) scores for the compounds were calculated using SwissADME, a free web tool that predicts synthetic feasibility based on fragmental analysis of molecular structures (https://www.swissadme.ch, accessed on 1 August 2025). The method estimates the ease of synthesis by analyzing the frequency of molecular fragments found in a large database of commercially available compounds. Fragment contributions are modulated by factors related to molecular size and complexity, such as the presence of chiral centers, spiro atoms, and macrocycles. The SA score is normalized on a scale from 1 (very easy to synthesize) to 10 (very difficult to synthesize), providing a rapid and reliable estimation of synthetic tractability suitable for large compound libraries.

### 3.2. QSAR Study of BChE Inhibition

To better understand the molecular factors influencing the butyrylcholinesterase (BChE) inhibitory activity of the synthesized pyrrole derivatives, a quantitative structure–activity relationship (QSAR) study was carried out on 15 compounds using the QSARINS software (version 2.24) [47].

Molecular descriptors were calculated with PaDEL-Descriptor [48], generating over 800 structural and physicochemical parameters. Descriptors with constant values (>95%) or high intercorrelation (|r| > 0.90) were removed to reduce redundancy and avoid multicollinearity.

The Genetic Algorithm–Multiple Linear Regression (GA-MLR) method in QSARINS was then used for descriptor selection and model building [49]. The dataset was randomly divided into 80% for training and 20% for external validation to ensure an unbiased assessment of predictive ability.

Model quality was evaluated using R^2^, R^2^adj, Q^2^, and R^2^test values. Parameters above 0.6 indicate good stability and predictive reliability [50]. Y-randomization and applicability domain analyses were also performed to confirm the absence of chance correlation and to define the reliability range of the model [51].

### 3.3. Synthesis

The solvents and reagents used in the experiments were sourced commercially from Sigma-Aldrich (St. Louis, MO, USA) and were utilized as received without further purification. The microwave-assisted chemical syntheses were performed using a FlexiWave Milestone Lab Microwave reactor equipped with fiber optic and infrared sensors for precise control. Reaction progress was monitored by thin-layer chromatography (TLC) using aluminum plates coated with silica gel and a chloroform–ethanol mixture (1:0.8) as the mobile phase. Melting points of the synthesized compounds were determined on a Kruss M5000 apparatus (KRÜSS, Hamburg, Germany). Infrared spectra spanning 4000 to 400 cm^−1^ were recorded with a Nicolet iS10 FT-IR spectrometer (Thermo Fisher Scientific, MA, USA) fitted with a Smart iTR accessory. Proton nuclear magnetic resonance (^1^H-NMR) spectra were obtained on a Bruker Avance Neo 400 MHz spectrometer (Biospin GmbH, Baden-Württemberg, Germany) using tetramethylsilane (TMS) as the internal reference standard. Mass spectrometry analyses were conducted using an Agilent 6410 LC-MS (Agilent Technologies, Santa Clara, CA, USA) triple quadrupole instrument employing the electrospray ionization (ESI) technique.

The pyrrole-based compounds (**EM-DC-1** to **EM-DC-26**) [27,28,29,30,31,32,33,34,35] were synthesized by reacting 0.1 mol of 2,5-hexanedione with 0.12 equivalents of the corresponding amino derivative. The dicarbonyl compound was first dissolved in a minimal amount of glacial acetic acid, followed by the addition of the aniline derivatives. The synthesis was performed using microwave irradiation in a specialized reactor that precisely controls the temperature and reaction time.

4-(2,5-dimethyl-1*H*-pyrrol-1-yl)phenol (**EM-DC-1**)

Solid; Yield 91%; Melting point 39.5–41.2 °C; IR *v*max: 2987 (O-H), 1650 (C=C), 1509 (aromatic ring), 1369 (C-N); ^1^H NMR (DMSO, 400 MHz): *δ* 2.01 (6H, s, CH_3_), 5.84 (2H, s, pyrrole ring), 7.11 (2H, d, *J* = 6.90 Hz, Ar), 7.56 (2H, d, *J* = 7.10 Hz, Ar), 10.19 (1H, s, -OH); *m*/*z* (FTMS + pESI) 188.10

4-(2,5-dimethyl-1*H*-pyrrol-1-yl)benzoic acid (**EM-DC-2**)

Solid; Yield 85%; Melting point 181.0–182.0 °C; IR *v*max: broad 2980 (O-H), 1687 (C=O), 1605 (C=C), 1318 (C-N);1283 (C-O); ^1^H NMR (DMSO, 400 MHz): *δ* 2.01 (6H, s, CH_3_), 5.84 (2H, s, pyrrole ring), 7.94 (2H, d, *J* = 7.60 Hz, Ar), 8.31 (2H, d, *J* = 7.40 Hz, Ar), 12.71 (1H, s, -OH); *m*/*z* (FTMS + pESI) 216.10

1-(2-bromophenyl)-2,5-dimethyl-1*H*-pyrrole (**EM-DC-3**)

Solid; Yield 83%; Melting point 75–77 °C; IR *v*max: 3070 (C-H), 1632 (C=C), 840 (C-Br); ^1^H NMR (DMSO, 400 MHz): *δ* 1.91 (6H, s, CH_3_), 5.83 (2H, s, pyrrole ring), 7.38–7.68 (4H, m, Ar); *m*/*z* (FTMS + pESI) 250.02

1-(4-bromophenyl)-2,5-dimethyl-1*H*-pyrrole (**EM-DC-4**)

Solid; Yield 84%; Melting point 67.5–69.3 °C; IR *v*max: 3070 (C-H), 1587 (C=C), 547 (C-Br); ^1^H NMR (DMSO, 400 MHz): *δ* 2.01 (6H, s, CH_3_), 5.84 (2H, s, pyrrole ring), 7.62 (2H, d, *J* = 7.30 Hz, Ar), 7.68 (2H, d, *J* = 7.50 Hz, Ar); *m*/*z* (FTMS + pESI) 250.02

1-(2-methoxy-5-methylphenyl)-2,5-dimethyl-1*H*-pyrrole (**EM-DC-5**)

Solid; Yield 79%; Melting point 62.5–63 °C; IR *v*max: 2950 (C-H), 1509 (C=C), 1258 (C-N); ^1^H NMR (DMSO, 400 MHz): *δ* 1.91 (6H, s, CH_3_), 2.45 (3H, s), 3.92 (3H, s), 5.77 (2H, s, pyrrole ring), 6.06 (2H, s), 7.05–7.76 (3H, m, Ar); *m*/*z* (FTMS + pESI) 216.13

1-(2*H*-1,3-benzodioxol-5-yl)-2,5-dimethyl-1*H*-pyrrole (**EM-DC-6**)

Solid; Yield 80%; Melting point 79.1–80 °C; IR *v*max: 2950 (C-H), 1545 (C=C), 1270 (C-O-C), 1221 (C-N); ^1^H NMR (DMSO, 400 MHz): *δ* 2.01 (6H, s, CH_3_), 5.84 (2H, s, pyrrole ring), 6.06 (2H, s), 7.00–7.41 (3H, m, Ar); *m*/*z* (FTMS + pESI) 216.10

2,5-dimethyl-1-(2-phenylethyl)-1*H*-pyrrole (**EM-DC-7**)

Oil; Yield 78%; IR *v*max: 3070 (CH, Aromatic), 2971 (C-H), 1600 (C=C), 1392 (C-N); ^1^H NMR (DMSO, 400 MHz): *δ* 2.19 (6H, s, CH_3_), 2.82 (t, J = 7.5 Hz, 2H), 4.18 (t, J = 7.5 Hz, 2H), 5.78 (2H, s, pyrrole ring), 7.19–7.23 (5H, m, Ar); *m*/*z* (FTMS + pESI) 200.14

1-[2-(4-chlorophenyl)ethyl]-2,5-dimethyl-1*H*-pyrrole (**EM-DC-8**)

Oil; Yield 81%; IR *v*max: 3306 (C-H), 2929 (C-H, aliphatic), 1637 (C=C), 1406 (C-N); 758 (C-Cl); ^1^H NMR (DMSO, 400 MHz): *δ* 2.19 (6H, s, CH_3_), 2.82 (2H, t, *J =* 7.40 Hz), 4.18 (2H, t, *J =* 7.80 Hz), 5.78 (2H, s, pyrrole ring), 7.14 (2H, d, *J* = 7.25 Hz, Ar), 7.35 (2H, d, *J* = 7.62 Hz, Ar); *m*/*z* (FTMS + pESI) 234.10

4-[2-(2,5-dimethyl-1*H*-pyrrol-1-yl)ethyl]phenol (**EM-DC-9**)

Solid; Yield 89%; Melting point 39–41 °C; IR *v*max: broad 3169 (O-H), 1612 (C=C), 1227 (C-O); ^1^H NMR (DMSO, 400 MHz): *δ* 2.19 (6H, s, CH_3_), 2.82 (2H, t, *J =* 7.40 Hz), 4.18 (2H, t, *J =* 7.80 Hz), 5.78 (2H, s, pyrrole ring), 6.68 (2H, d, *J* = 7.65 Hz, Ar), 6.96 (2H, d, *J* = 7.25 Hz, Ar), 9.06 (1H, s, -OH); *m*/*z* (FTMS + pESI) 216.13

4-[2-(2,5-dimethyl-1*H*-pyrrol-1-yl)-1-hydroxyethyl]phenol (**EM-DC-10**)

Solid; Yield 92%; Melting point 39–41 °C; IR *v*max: 3055 (O-H), 1590 (C=C), 1319 (C-N), 1250 (C-O); ^1^H NMR (DMSO, 400 MHz): *δ* 2.20 (6H, s, CH_3_), 3.20 (2H, d, J = 7.6 Hz), 4.25 (1H, t, J = 7.8 Hz), 5.20 (d, J = 7.1 Hz), 5.80 (2H, s, pyrrole ring), 6.71 (2H, d, J = 7.7 Hz, Ar H), 7.10 (2H, d, J = 7.3 Hz, Ar H), 9.05 (1H, s, -OH); *m*/*z* (FTMS + pESI) 232.13

4-[1-(2,5-dimethyl-1*H*-pyrrol-1-yl)propan-2-yl]phenol (**EM-DC-11**)

Solid; Yield 83%; Melting point 48–49 °C; IR *v*max: 3160 (O-H), 1575 (C=C), 1360 (C-N), 1240 (C-O); ^1^H NMR (DMSO, 400 MHz): *δ* 1.25 (3H, s, CH_3_), 2.19 (6H, s, CH_3_), 3.50 (2H, d, J = 7.4 Hz), 4.25 (1H, t, J = 7.5 Hz), 5.78 (2H, s, pyrrole ring), 6.71 (2H, d, J = 7.7 Hz, Ar H), 7.10 (2H, d, J = 7.3 Hz, Ar H), 9.05 (1H, s, -OH); *m*/*z* (FTMS + pESI) 230.15

4-(2,5-dimethyl-1*H*-pyrrol-1-yl)pyridine (**EM-DC-12**)

Solid; Yield 91%; Melting point 72–73 °C; IR *v*max: 1615 (C=C), 1242 (C-N); ^1^H NMR (DMSO, 400 MHz): *δ* 2.00 (6H, s, CH_3_), 5.80 (2H, s, pyrrole ring), 7.50 (2H, d, *J* = 7.5 Hz, Ar H), 8.56 (2H, d, J = 7.5 Hz, Ar H); *m*/*z* (FTMS + pESI) 173.07

4-(2,5-dimethyl-1*H*-pyrrol-1-yl)-2-methylquinoline (**EM-DC-13**)

Solid; Yield 83%; Melting point 101–102.5 °C; IR *v*max: 2944 (N-C),1513 (C=C), 1419 (C=N), 1282 (C-N); ^1^H NMR (DMSO, 400 MHz): *δ* 1.90 (6H, s, CH_3_), 2.74 (3H, s, CH_3_), 5.80 (2H, s, pyrrole ring), 7.80–8.25 (m, 5H, quinoline ring); *m*/*z* (FTMS + pESI) 237.13

2-(2,5-dimethyl-1*H*-pyrrol-1-yl)-2-methylpropan-1-ol (**EM-DC-14**)

Oil; Yield 76%; IR *v*max: 3105 (O-H), 1450 (C=C), 1260 (C-N); ^1^H NMR (DMSO, 400 MHz): *δ* 1.20 (6H, s, CH_3_), 2.20 (6H, s, CH_3_), 3.55 (2H, s), 5.30 (1H, s, -OH), 5.80 (2H, s, pyrrole ring); *m*/*z* (FTMS + pESI) 168.13

2-(2,5-dimethyl-1*H*-pyrrol-1-yl)-3-sulfanylpropanoic acid (**EM-DC-15**)

Solid; Yield 91%; Melting point 96–98 °C; IR *v*max: 3200 (O-H), 2540 (S-H), 1420 (C-N); ^1^H NMR (DMSO, 400 MHz): *δ* 1.25 (1H, s, SH), 2.20 (6H, s, CH_3_), 3.19 (2H, m), 3.55 (2H, s), 4.85 (1H, t, *J* = 7.40 Hz), 5.80 (2H, s, pyrrole ring), 11.30 (1H, s, -OH); *m*/*z* (FTMS + pESI) 200.07

4-[2-(2,5-dimethyl-1*H*-pyrrol-1-yl)ethyl]morpholine (**EM-DC-16**)

Oil; Yield 82%; IR *v*max: 3150 (N-H), 1425 (C=C), 1320 (C-N); ^1^H NMR (DMSO, 400 MHz): *δ* 2.20 (6H, s, CH_3_), 2.36 (4H, t, *J* = 7.5 Hz), 2.61 (2H, t, *J* = 7.40 Hz), 3.56 (4H, t, *J* = 7.45 Hz), 4.52 (2H, t, *J* = 7.5 Hz), 5.80 (2H, s, pyrrole ring); *m*/*z* (FTMS + pESI) 209.16

4-(2,5-dimethyl-1*H*-pyrrol-1-yl)-3-hydroxybenzoic acid (**EM-DC-17**)

Solid; Yield 93%; Melting point 165–167 °C; IR *v*max: 3350 (O-H), 3150 (N-H), 1710 (C=O), 1520 (C=C), 1250 (C-O); ^1^H NMR (DMSO, 400 MHz): *δ* 1.90 (6H, s, CH_3_), 5.80 (2H, s, pyrrole ring), 7.66–7.87 (3H, m, Ar), 10.01 (1H, s, -OH), 12.70 (1H, s, -OH); *m*/*z* (FTMS + pESI) 232.09

5-(2,5-dimethyl-1*H*-pyrrol-1-yl)-2-hydroxybenzoic acid (**EM-DC-18**)

Solid; Yield 93%; Melting point 169–171 °C; IR *v*max: 3214 (O-H), 3038 (N-H), 1685 (C=O), 1460 (C=C); ^1^H NMR (DMSO, 400 MHz): *δ* 2.01 (6H, s, CH_3_), 5.84 (2H, s, pyrrole ring), 7.16 (1H, d, Ar), 7.90 (1H, d, Ar), 8.97 (1H, s, Ar), 12.04 (1H, s, -OH), 14.25 (1H, s, -OH); *m*/*z* (FTMS + pESI) 232.09

2-(2,5-dimethyl-1*H*-pyrrol-1-yl)-3-(2*H*-imidazol-4-yl)propanoic acid (**EM-DC-19**)

Solid; Yield 87%; Melting point 111–113 °C; IR *v*max: 3204 (N-H), 3055 (O-H), 1705 (C=O); ^1^H NMR (DMSO, 400 MHz): *δ* 1.30 (2H, s), 2.20 (6H, s, CH_3_), 2.75 (2H, d), 4.60 (1H, t, *J* = 7.6 Hz), 5.78 (2H, s, pyrrole ring), 8.45 (1H, s, imidazole), 12.22 (1H, s, -OH); *m*/*z* (FTMS + pESI) 234.12

2-(2,5-dimethyl-1*H*-pyrrol-1-yl)-3-(4-hydroxyphenyl)propanoic acid (**EM-DC-20**)

Solid; Yield 92%; Melting point 153–155 °C; IR *v*max: 3320 (O-H), 1680 (C=O); ^1^H NMR (DMSO, 400 MHz): *δ* 2.20 (6H, s, CH_3_), 3.17 (2H, d), 4.95 (1H, t, *J* = 7.5 Hz), 5.78 (2H, s, pyrrole ring), 6.68–6.96 (4H, m, Ar), 9.06 (1H, s, -OH), 12.22 (1H, s, -OH); *m*/*z* (FTMS + pESI) 260.12

2-(2,5-dimethyl-1*H*-pyrrol-1-yl)butanoic acid (**EM-DC-21**)

Solid; Yield 93%; Melting point 92.9–93.5 °C; IR *v*max: 3010 (O-H), 1690 (C=O), 1450 (C-O); ^1^H NMR (DMSO, 400 MHz): *δ* 0.94 (3H, t, *J =* 7.4 Hz), 1.83 (2H, m), 2.20 (6H, s, CH_3_), 4.47 (1H, t, *J =* 7.30 Hz), 5.78 (2H, s, pyrrole ring), 12.20 (1H, s, -OH); *m*/*z* (FTMS + pESI) 182.11

2-(2,5-dimethyl-1*H*-pyrrol-1-yl)-3-hydroxypropanoic acid (**EM-DC-22**)

Solid; Yield 86%; Melting point 76.5–77 °C; IR *v*max: 2970 (O-H), 1718 (C=O), 1630 (C=C), 1200 (C-O); ^1^H NMR (DMSO, 400 MHz): *δ* 2.20 (6H, s, CH_3_), 4.15 (2H, m), 4.57 (1H, t, *J =* 7.80 Hz), 5.78 (2H, s, pyrrole ring), 6.35 (1H, s, -OH), 12.20 (1H, s, -OH); *m*/*z* (FTMS + pESI) 184.09

3-(3,4-dihydroxyphenyl)-2-(2,5-dimethyl-1*H*-pyrrol-1-yl)propanoicacid (**EM-DC-23**)

Solid; Yield 90%; Melting point 143–145 °C; IR *v*max: 3150 (O-H), 1705 (C=O), 1490 (C=C), 1240 (C-O); ^1^H NMR (DMSO, 400 MHz): *δ* 2.20 (6H, s, CH_3_), 3.17 (2H, m), 4.95 (1H, t, *J =* 7.80 Hz), 5.78 (2H, s, pyrrole ring), 6.52–6.61 (3H, m, Ar), 9.48 (2H, s, -OH), 12.72 (1H, s, -OH); *m*/*z* (FTMS + pESI) 276.12

[4-(2,5-dimethyl-1*H*-pyrrol-1-yl)benzamido]acetic acid (**EM-DC-24**)

Solid; Yield 83%; Melting point 164–165 °C; IR *v*max: 3240 (N-H), 1685 (C=O); ^1^H NMR (DMSO, 400 MHz): *δ* 2.01 (6H, s, CH_3_), 3.84 (2H, s), 5.84 (2H, s, pyrrole ring), 7.71 (2H, d, Ar), 8.13 (2H, d, Ar), 13.03 (1H, s, -OH); *m*/*z* (FTMS + pESI) 273.12

1-benzyl-4-(2,5-dimethyl-1*H*-pyrrol-1-yl)piperidine (**EM-DC-25**)

Oil; Yield 78%; IR *v*max: 3250 (N-H), 1535 (C=C), 1480 (C=N); ^1^H NMR (DMSO, 400 MHz): *δ* 1.85–2.41 (4H, m, piperidine), 2.20 (6H, s, CH_3_), 3.70 (2H, s), 3.75 (1H, m), 5.78 (2H, s, pyrrole ring), 7.21–7.29 (5H, m, Ar); *m*/*z* (FTMS + pESI) 269.20

2,5-dimethyl-1-(2-methylbutyl)-1*H*-pyrrole (**EM-DC-26**)

Oil; Yield 74%; IR *v*max: 3350 (N-H), 1610 (C=C), 1520 (C=N); ^1^H NMR (DMSO, 400 MHz): *δ* 0.93 (3H, d, *J =* 7.40 Hz), 0.99 (3H, t, *J =* 7.80 Hz), 1.55 (2H, m), 1.50 (1H, m), 2.20 (6H, s, CH_3_), 3.90 (2H, m), 5.78 (2H, s, pyrrole ring); *m*/*z* (FTMS + pESI) 166.15

[4-(2,5-dimethyl-1*H*-pyrrol-1-yl)phenyl]acetic acid (**EM-DC-27**)

Solid; Yield 91%; Melting point 122–124 °C; IR *v*max: 3050 (O-H), 1690 (C=O), 1580 (C=C), 1420 (C-N); ^1^H NMR (DMSO, 400 MHz): *δ* 2.01 (6H, s, CH_3_), 3.69 (2H, s), 5.84 (2H, s, pyrrole ring), 7.29 (2H, d, Ar), 7.58 (2H, d, Ar), 12.39 (1H, s, -OH); *m*/*z* (FTMS + pESI) 230.11

### 3.4. In Vitro Enzymatic Activity Evaluation of AChE, BChE, MAO-A, and MAO-B

Monoamine oxidase (MAO) activity for both recombinant human MAO-A (*h*MAOA) and MAO-B (*h*MAOB) was assessed using a fluorometric assay based on the Amplex^®^ UltraRed reagent. This method, originally reported by Bautista-Aguilera et al. [52] and later adapted with minor changes by Kasabova-Angelova et al. [53], detects hydrogen peroxide or peroxidase activity present in enzymes or biological samples. In this assay, Amplex^®^ Red reacts in a 1:1 molar ratio with hydrogen peroxide in the presence of peroxidase, producing the red-fluorescent compound resorufin. Due to resorufin’s strong extinction coefficient (58,000 ± 5000 cm^−1^·M^−1^), measurements could be accurately taken using either fluorometric or spectrophotometric methods. This setup permits detection of extremely low hydrogen peroxide concentrations, down to the picomolar range (approximately 10 pM in a 100 µL volume). The control conditions included the use of purified *h*MAOA or *h*MAOB in reaction buffer, enzyme solutions with hydrogen peroxide, and buffer alone. All the test compounds were initially prepared at a 1 µM final concentration. These compounds were combined with either *h*MAOA or *h*MAOB and loaded into a 96-well plate (with eight replicates per test compound), followed by incubation for 30 min at 37 °C in the dark. Subsequently, a 50 µL detection mix was added to each well. This mix contained Amplex^®^ Red, horseradish peroxidase (HRP), and tyramine substrate, all prepared in a reaction buffer. As this enzymatic reaction proceeded continuously, the fluorescence signal was recorded every 30 min (at 0, 30, 60, 90, 120, and 150 min) to track enzymatic kinetics. Readings were taken in dark conditions while the plates were agitated and maintained at 37 °C. Fluorescence was measured using a Synergy 2 Microplate Reader at wavelengths of 570 nm and 690 nm.

AChE and BChE inhibitory activity of the newly synthesized pyrrole-based compounds was measured using the microplate assay described by Ellman et al. [54] with the modifications added by López et al. [55]. The compounds were tested at concentrations between 0.01 and 200 μM. First, they were dissolved in phosphate buffer (PBS) (8 mM K_2_HPO_4_, 2.3 mM NaH_2_PO_4_, 0.15 M NaCl, pH 7.5), assisted by the addition of no more than 5% MeOH. Then, they were serially diluted with PBS to provide the concentration range needed. Acetylcholinesterase from *Electrophorus electricus* and butyrylcholinesterase from equine serum (Sigma-Aldrich, Hamburg, Germany) were used with a substrate solution of 5,5′-dithiobis(2-nitrobenzoic acid) (DTNB) with acetylthiocholine iodide (ATCI) or butyrylthiocholine iodide (BTCI), respectively (0.04 M Na_2_HPO_4_, 0.2 mM DTNB, 0.24 mM ATCI or BTCI, pH 7.5). A total of 50 μL of AChE or BChE (0.25 U/mL, in PBS) and 50 μL of the tested compound solution were added to the wells. Incubation of the plates was performed at room temperature for 30 min. Then, 100 μL of substrate solution was added to start the enzymatic reaction. The absorbances were read in a microplate reader (BIOBASE, ELISA-EL10A, Jinan, China) at 405 nm after 5 min for AChE and 10 min for BChE. Enzyme activity was calculated as an inhibition percentage compared to an assay including buffer instead of an inhibitor. Galanthamine and donepezil were used as positive controls.

### 3.5. Animals

A total of 10 Male Wistar rats with a body weight of 200–250 g were used. They were housed in plexiglass cages (3 per cage) in a 12/12 light/dark cycle under standard laboratory conditions (ambient temperature 20 °C ± 2 °C and humidity 72% ± 4%) with free access to water and standard pelleted rat food 53-3, produced according to ISO 9001:2008 [56]. The animals were purchased from the National Breeding Centre, Sofia, Bulgaria. Seven days’ acclimatization was allowed before the commencement of the study, and a veterinary physician monitored the health of the animals regularly. Vivarium (certificate of registration of farm № 0072/01.08.2007) was inspected by the Bulgarian Drug Agency in order to check the husbandry conditions (№A-11-1081/03.11.2011).

All the performed procedures were approved by the Institutional Animal Care Committee of the Medical University of Sofia (Bulgarian Agency for Food Safety with Permission № 190, approved on February 6, 2020, and valid till 2026). The principles stated in the European Convention for the Protection of Vertebrate Animals used for Experimental and other Scientific Purposes (ETS 123) were strictly followed throughout the experiment. The brains were taken from the rats immediately after decapitation and stored on ice until needed.

### 3.6. Sub-Cellular in Vitro Studies

Two types of buffers were used for the isolation of the rat brain synaptosomes and mitochondria. Buffer A was 5 mM HEPES and 0.32 M Sucrose; Buffer B was 290 mM NaCl, 0.95 mM MgCl_2_ × 2H2O, 10 mM KCl, 2.4 mM CaCl_2_ × 2H2O, 2.1 mM NaH_2_PO_4_, 44 mM HEPES, 13 mM D-Glucose. For the gradient centrifugation, Percoll reagent was used. A stock solution of 90% Percoll was used to prepare 16%, 10% and 7.5% solutions. All the solutions were used as described below. Immediately after harvesting, synaptosomes, microsomes, and mitochondria were incubated with the exam compounds for 1 h. For assessing the possible neurotoxicity of the most MAOB active compounds, the sub-cellular fractions were incubated with 100 µM of them, and for evaluating their possible neuroprotective and antioxidant activities in different models of neurotoxicity, the sub-cellular fractions were incubated with 50µM of them. The protein measurement of all the sub-cellular fractions was made by the method of Lowry et al., 1951 [57].

#### 3.6.1. Rat Brain Synaptosomes—Isolation and Incubation

The synaptosomes were prepared from rat brains by multiple sub-cellular fractionation using a Percoll gradient [58].

#### 3.6.2. Model of 6-OHDA-Induced Neurotoxicity

This in vitro model resembles the neurodegenerative processes occurring in PD. Dopamine metabolism and oxidation led to the formation of reactive oxygen species (ROS) and reactive quinones. These processes were catalyzed by the MAOB enzyme. They induced dopamine neurotoxicity and neurodegeneration. The synaptosomes were incubated with 150 μM 6-OHDA for 1 h.

#### 3.6.3. Synaptosomal Viability

After the incubation, the synaptosomes were centrifuged three times at 15,000× *g* for 1 min. MTT-test was performed to determine synaptosomal vitality by the method described by Mungarro-Menchaca et al. [59].

#### 3.6.4. Determination of Reduced Glutathione (GSH)

The level of GSH was determined by measuring the non-protein SH-groups after precipitation of the proteins with trichloroacetic acid (TCA). The presence of thiols in the supernatant was determined using Elmman’s reagent. The resulting yellow color was measured spectrophotometrically (λ = 412 nm).

#### 3.6.5. Rat Brain Microsomes—Preparation

The brain was homogenized in 9 vol. of 0.1 M Tris buffer containing 0.1 mM Dithiothreitol, 0.1 mM Phenylmethylsulfonyl fluoride, 0.2 mM EDTA, 1.15% KCl, and 20% (*v*/*v*) glycerol at pH 7.4. The homogenate was centrifuged twice at 17,000× *g* for 30 min. The supernatants from both centrifugations were combined and centrifuged twice at 100,000× *g* for 1 h. The resulting pellet was frozen in the Tris until needed [60].

#### 3.6.6. FeSO_4_/Ascorbic Acid-Induced Lipid Peroxidation (LPO)

The LPO was started with a solution of 20 μM iron sulfate and 0.5 mM ascorbic acid [61].

#### 3.6.7. Malondialdehyde (MDA) Assay

The quantity of the lipid peroxidation product MDA was assessed using the method described by Deby and Goutier [62].

#### 3.6.8. Rat Brain Mitochondria—Isolation

The mitochondria were prepared by multiple differential fractionation using a Percoll gradient [63].

#### 3.6.9. Tert-Butyl Hydroperoxide (t-BuOOH)-Induced Oxidative Stress

The mitochondria were incubated with 75 μM *t*-BuOOH [57].

#### 3.6.10. Lipid Peroxidation Assay

After incubation of the mitochondria, 0.3 mL 0.2% TBA and 0.25 mL sulfuric acid (0.05 M) were added to stop the reaction. The tubes were maintained at 100 °C for 30 min. In the next stage, centrifugation of the tubes was carried out at 3500× *g* for 10 min. Assessment of the total quantity of MDA formed in each sample was measured in the supernatant at 532 nm [64].

#### 3.6.11. Measurement of GSH Content

After incubation of mitochondria, 0.04% DTNB was mixed with the mitochondrial suspensions in 0.1 M phosphate buffer (pH 7.4). The absorbance of the yellow product was measured at 412 nm [65].

### 3.7. Statistical Analysis

The statistical analysis was performed using the Prism 9 software package (GraphPad Inc., San Diego, CA, USA). IC_50_ values, where applicable, were determined in triplicate, and the results were presented as mean values. Statistical analysis was performed by one-way analysis of variance (ANOVA) with post hoc multiple comparisons procedure (Dunnet’s test) to assess the statistical differences in the case of normal distribution. Values of *p* < 0.05, *p* < 0.01, and *p* < 0.001 were considered statistically significant. Statistical analysis of the results obtained from brain microsomes, synaptosomes, and mitochondria was performed using the statistical program “MEDCALC”. The results were expressed as mean ± SEM for six experiments. The significance of the data was assessed using the non-parametric Mann–Whitney test. Values of *p* < 0.05; *p* < 0.01; and *p* < 0.001 were considered statistically significant.

### 3.8. In Silico Knowledge-Based Toxicity Prediction

Toxicity assessment was carried out using the Derek Nexus software (Knowledge Base v6.4.1; Nexus v2.7.2, Lhasa Limited, Leeds, UK). This tool applies a rule-based methodology based on structural alerts derived from experimental toxicology data, the published literature, and regulatory sources. The leader compounds—**EM-DC-19** and **EM-DC-27**—were analyzed by entering their canonical SMILES code, generated by ChemDraw (version 25.0), into the Derek Nexus platform. The analysis covered all the available toxicity endpoints, including carcinogenicity, genotoxicity, organ-specific toxicity, reproductive and developmental effects, sensitization, phototoxicity, and phospholipidosis. Predictions were generated for both bacterial systems (relevant to the Ames assay) and mammalian models (rat and human). According to the program’s criteria, each prediction was categorized as probable, plausible, equivocal, or negative.

## 4. Conclusions

This study employed an integrative consensus docking approach combining Glide, GOLD 5.3, and MM/GBSA rescoring to identify MAO-B inhibitors with additional AChE inhibition from a large pyrrole-based compound library. The validated protocols achieved high enrichment values and hit rates. Experimental validation confirmed potent inhibition by the selected hits, including **EM-DC-19** and **EM-DC-27**, alongside favorable neuroprotective and antioxidant effects, supporting their potential relevance in neurodegenerative disease therapy.

Future work will pursue focused chemical modifications to enhance blood–brain barrier penetration and selectivity, particularly for **EM-DC-27**, alongside advancing **EM-DC-19** towards preclinical evaluation. Additionally, comprehensive in vivo studies and mechanistic investigations of neuroprotective pathways are required to validate their clinical potential. This research opens avenues for novel multitarget therapeutics for neurodegenerative diseases, addressing both symptomatic relief and underlying neurodegeneration mechanisms.

## Figures and Tables

**Table 1:** Number of approved and rejected poses after consensus docking of 1% of the applied MAO-B (**A**) and AChE (**B**) datasets.

** 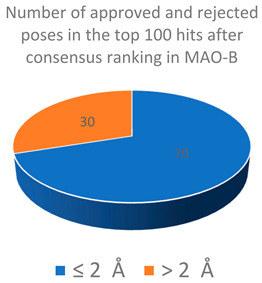 **	** 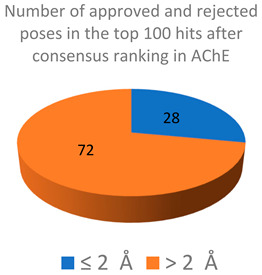 **
**A**	**B**

**Table 2:** Docking scores obtained from virtual screening of compounds against the enzymes MAO-B and AChE.

Compound	MAO-B (PDB: 2V5Z)		RMSD Value (Å)	AChE (PDB: 4EY6)		RMSD Value (Å)	Synthetic Accessibility (SA) *
	MM/GBSA (kcal/mol)	GOLD 5.3 (ChemPLP)		MM/GBSA (kcal/mol)	GOLD 5.3 (ChemPLP)		
EM-DC-1	−61.54	152.21	1.47	−52.28	110.31	1.25	1.50
EM-DC-2	−60.32	142.63	1.54	−53.43	123.26	1.22	1.55
EM-DC-3	−60.18	150.23	2.24	−50.53	112.53	1.63	1.75
EM-DC-4	−59.84	146.72	0.84	−53.28	106.77	1.58	1.64
EM-DC-5	−59.79	142.48	0.33	−48.45	102.73	0.71	1.90
EM-DC-6	−59.74	147.36	2.87	−54.67	107.54	1.52	2.34
EM-DC-7	−59.69	151.53	0.91	−55.48	111.92	2.42	1.57
EM-DC-8	−59.62	152.48	0.58	−52.81	122.68	1.52	1.72
EM-DC-9	−59.55	145.27	2.94	−54.23	105.65	1.33	1.60
EM-DC-10	−59.57	150.64	1.59	−51.51	115.51	2.03	2.27
EM-DC-11	−59.51	149.63	0.46	−47.28	100.57	1.62	2.15
EM-DC-12	−59.45	144.72	1.25	−53.59	107.48	2.73	1.57
EM-DC-13	−58.28	147.62	1.91	−57.92	101.83	1.04	2.00
EM-DC-14	−58.24	148.26	1.07	−58.54	103.54	2.43	1.49
EM-DC-15	−58.17	150.62	1.46	−48.56	112.34	1.63	2.26
EM-DC-16	−57.92	151.71	1.82	−48.37	108.45	1.34	2.03
EM-DC-17	−57.84	148.64	0.87	−51.91	115.34	2.38	1.83
EM-DC-18	−57.83	149.13	2.64	−56.57	108.94	1.28	1.78
EM-DC-19	−57.76	150.27	1.57	−55.43	122.45	1.36	2.94
EM-DC-20	−57.72	148.60	1.44	−54.41	107.94	1.72	2.42
EM-DC-21	−52.37	116.22	1.67	−43.20	103.47	2.53	2.04
EM-DC-22	−51.32	112.67	0.84	−53.13	122.15	1.48	2.12
EM-DC-23	−54.02	120.31	0.67	−47.27	104.37	2.23	2.56
EM-DC-24	−53.41	112.64	1.31	−48.85	121.84	1.08	1.79
EM-DC-25	−53.92	113.79	0.83	−51.54	114.15	1.48	2.75
EM-DC-26	−50.28	121.37	2.74	−47.16	111.28	1.57	1.76
EM-DC-27	−54.24	138.82	1.94	−43.81	101.28	2.82	1.71
Galanthamine	n/a	n/a	n/a	−62.58	121.25	1.08	
Donepezil	n/a	n/a	n/a	−83.76	154.81	0.84	
Selegiline	−55.28	144.51	0.47	n/a	n/a	n/a	

n/a—not available. * synthetic accessibility (SA) scores calculated by SwissADME range from 1 to 10, where 1 indicates compounds that are very easy to synthesize and 10 indicates compounds that are very difficult to synthesize.

**Table 3:** Inhibitory activities and IC_50_ values of the top-ranked compounds against AChE, BChE, MAO-A, and MAO-B.

Compound	Inhibition Activity 200 µM AchE ^a^	AChE IC_50_ (µM) ^b^	Inhibition Activity 200 µM BChE ^a^	BChE IC_50_ (µM) ^b^	Inhibition Activity 1 µM MAO-A ^a^	MAO-A IC_50_ (µM) ^b^	Inhibition Activity 1 µM MAO-B ^a^	MAO-B IC_50_ (µM) ^b^
EM-DC-1	7.52 ± 2.07	>200	53.93 ± 2.14	172.70 ± 7.30	55 ± 7.1	0.32 ± 0.10	99 ± 7.1	>100
EM-DC-2	93.60 ± 4.66	0.75 ± 0.06	57.25 ± 0.58	158.06 ± 11.76	75 ± 6.9	0.43 ± 0.10	75 ± 7.1	0.44 ± 0.10
EM-DC-3	8.32 ± 2.64	>200	0.04 ± 9.01	>200	70 ± 7.2	0.52 ± 0.10	75 ± 7.1	0.56 ± 0.10
EM-DC-4	1.40 ± 0.38	>200	0	>200	60 ± 6.8	0.32 ± 0.10	98 ± 7.1	>100
EM-DC-5	39.78 ± 2.64	280.46 ± 21.27	74.02 ± 1.56	73.81 ± 5.96	98 ± 7.3	>100	99 ± 7.1	>100
EM-DC-6	64.98 ± 1.14	128.30 ± 1.87	36.26 ± 3.54	330.20 ± 59.77	97 ± 7.1	>100	98 ± 7.1	>100
EM-DC-7	11.90 ± 2.33	>200	43.86 ± 1.70	224.26 ± 8.12	80 ± 7.2	0.51 ± 0.10	75 ± 7.1	0.49 ± 0.10
EM-DC-8	31.12 ± 2.48	455.33 ± 80.71	35.42 ± 2.91	259.73 ± 13.47	99 ± 7.3	>100	99 ± 7.1	>100
EM-DC-9	72.86 ± 3.02	76.84 ± 4.68	73.70 ± 4.50	69.51 ± 7.52	98 ± 6.8	>100	98 ± 7.1	>100
EM-DC-10	60.35 ± 1.05	147.53 ± 5.91	87.17 ± 1.60	11.33 ± 0.87	99 ± 6.8	>100	99 ± 7.1	>100
EM-DC-11	29.00 ± 2.47	306.70 ± 13.71	30.56 ± 2.11	425.70 ± 54.80	97 ± 6.9	>100	97 ± 7.1	>100
EM-DC-12	65.69 ± 3.25	102.57 ± 5.85	73.25 ± 1.80	72.79 ± 2.53	70 ± 6.9	0.49 ± 0.10	70 ± 7.1	0.51 ± 0.10
EM-DC-13	91.15 ± 1.22	3.81 ± 0.26	83.81 ± 0.67	10.52 ± 0.17	80 ± 7.1	0.38 ± 0.10	80 ± 7.1	0.41 ± 0.10
EM-DC-14	33.24 ± 2.03	362.70 ± 48.63	0	>200	75 ± 7.1	0.44 ± 0.10	75 ± 7.1	0.46 ± 0.10
EM-DC-15	63.86 ± 1.29	130.83 ± 1.85	19.06 ± 2.47	>200	70 ± 7.2	0.55 ± 0.10	70 ± 7.1	0.53 ± 0.10
EM-DC-16	85.26 ± 2.43	11.79 ± 1.10	48.75 ± 1.50	204.83 ± 9.62	80 ± 7.3	0.46 ± 0.10	80 ± 7.1	0.47 ± 0.10
EM-DC-17	0	>200	3.85 ± 2.99	>200	99 ± 6.6	>100	99 ± 7.1	>100
EM-DC-18	15.99 ± 1.40	>200	0	>200	98 ± 6.5	>100	98 ± 7.1	>100
**EM-DC-19**	**73.98 ± 0.59**	**76.15 ± 6.12**	**18.03 ± 0.17**	**>200**	**99 ± 6.1**	**>100**	**50 ± 7.1**	**0.29 ± 0.10**
EM-DC-20	0	>200	0	>200	75 ± 6.4	0.60 ± 0.10	75 ± 7.1	0.62 ± 0.10
EM-DC-21	0	>200	0	>200	99 ± 7.1	>100	99 ± 7.1	>100
EM-DC-22	0	>200	0	>200	99 ± 7.2	>100	99 ± 7.1	>100
EM-DC-23	21.81 ± 2.14	480.03 ± 26.56	0	>200	98 ± 7.3	>100	98 ± 7.1	>100
EM-DC-24	24.08 ± 4.10	348.80 ± 25.17	0	>200	80 ± 7.1	0.58 ± 0.10	80 ± 7.1	0.59 ± 0.10
EM-DC-25	74.76 ± 1.47	87.19 ± 5.70	69.76 ± 0.81	103.93 ± 2.50	60 ± 7.1	0.33 ± 0.10	98 ± 7.1	>100
EM-DC-26	12.84 ± 3.54	>200	68.25 ± 1.60	106.50 ± 0.70	97 ± 7.1	>100	97 ± 7.1	>100
**EM-DC-27**	**26.46 ± 2.36**	**375.20 ± 52.99**	**0**	**>200**	**99 ± 7.1**	**>100**	**50 ± 7.1**	**0.34 ± 0.10**
Galanthamine	93.24 ± 0.52	1.31 ± 0.07	89.16 ± 0.44	26.62 ± 0.79	-	-	-	-
Donepezil	91.01 ± 0.53	0.0632 ± 0.0081	92.25 ± 0.22	6.88 ± 0.26	-	-	-	-
Selegiline	-	-	-	-	-	-	45%	0.32 ± 0.09
Chlorgyline	-	-	-	-	45 ± 6.6	0.33 ± 0.09	-	-

^a^ Inhibition percentage of AChE/BChE by 200 µM solution and for MAO-A/B, 1 µM of the tested compounds. ^b^ Inhibitor concentration (means ± SD of three experiments) for 50% inactivation of AChE/BChE/MAO-A/B.

**Table 4:** The developed model using GA-MLR and its evaluated statistical parameters.

Model Equation: pIC_50_ = 14.588 + 0.197 SHBint2 − 16.205 ETA_Epsilon_5 + 0.181 nAtomP				
R	0.930	Descriptor	VIF	T_test_
R^2^	0.867	SHBint2	1.840	5.291
Q^2^	0.735	ETA_Epsilon_5	1.842	−3.947
R^2^adj	0.830	nAtomP	1.004	6.872
R^2^ _test_	0.600			

**Table 5:** In silico ADME and toxicological properties of the lead compounds.

Compound	Mol Weight (a)	QLogP o/w (b)	QPlogBB (c)	PSA (d)	Rule of Five Violations (e)	Toxicological Alerts (f)
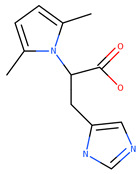	233.26	2.41	−0.624	68.38	0	0
**EM-DC-19**						
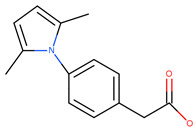	229.27	3.50	−0.451	51.91	0	1
**EM-DC-27**						

(a) Molecular weight (range: 130.0–725.0); (b) predicted octanol/water partition coefficient (range: −2.0 to 6.5); (c) predicted brain–blood partition coefficient (range: −3.0 to 1.2); (d) polar surface area of polar nitrogen and oxygen atoms (range: 7.0–200.0); (e) violations of Lipinski’s rule of five; (f) toxicological endpoints predicted by Derek Nexus.

## Data Availability

The original contributions presented in this study are included in the article/Supplementary Materials. Further inquiries can be directed to the corresponding author.

## Supplementary Materials

The following supporting information can be downloaded at https://www.mdpi.com/article/10.3390/ph18111677/s1. **Table S1:** Fragments used in the generation of the pyrrole-based database; **Table S2:** MM/GBSA Ranks of the active MAO-B inhibitors included in the observed dataset, together with the corresponding Specificity, 1-Specificity and Sensitivity; **Table S3:** MM/GBSA ranks of the active AChE inhibitors included in the observed dataset, together with the corresponding Specificity, 1-Specificity and Sensitivity; **Table S4:** RMSD values of the top ranked 100 solutions with MM/GBSA of the applied MAO-B database. The rejected solutions with RMSD over 2 angstroms were highlighted; **Table S5:** RMSD values of the top ranked 100 solutions with MM/GBSA of the applied AChE database. The rejected solutions with RMSD over 2 angstrioms were highlighted; **Figure S1.** Active conformations of compounds EM-DC-21 to EM-DC-27 in the MAO-B active site (PDB ID: 2V5Z); **Figure S2:** LC/MS, ^1^H-NMR, and IR spectra of the lead compounds **EM-DC-19** (panels A, B, C) and **EM-DC-27** (panels D, E, F); **Figure S3:** Active conformations of EM-DC-19 and EM-DC-27 in MAO-B and AChE enzymes; **Table S6**: Toxicity Endpoints Predicted by Derek Nexus.
