# Supplementary material for: Design, Synthesis, and Evaluation of Pyrrole-Based Selective MAO-B Inhibitors with Additional AChE Inhibitory and Neuroprotective Properties Identified via Virtual Screening
Source: Pharmaceuticals (Basel). 2025 Nov 5;18(11):1677. doi: 10.3390/ph18111677 (PMC12655551; doi:10.3390/ph18111677)
Supplement: Supplementary file 1 [file pharmaceuticals-18-01677-s001.zip › pharmaceuticals-3921271-supplementary.pdf]

Design, Synthesis, and Evaluation of Pyrrole-Based Selective MAO-B Inhibitors with Additional AChE Inhibitory and Neuroprotective Properties Identified via Virtual Screening

Emilio Mateev[1,*], Samir Chtita[2], Ekaterina Pavlova[3], Ali Irfan[4], Diana Tzankova[1], Shubham Sharma[5], Borislav Georgiev[6], Alexandrina Mateeva[1], Georgi Momekov[3], Maya Georgieva[1], Alexander Zlatkov[1], Magdalena Kondeva-Burdina[3*]

[1]Department of Pharmaceutical chemistry, Faculty of Pharmacy, Medical University – Sofia, Sofia 1000, Bulgaria;
[2]Laboratory of Analytical and Molecular Chemistry, Faculty of Sciences Ben M'Sik, Hassan II University of Casablanca, Morocco.
[3]Department of Pharmacology, pharmacotherapy and toxicology, Faculty of Pharmacy, Medical University – Sofia, Sofia 1000, Bulgaria
[4]Department of Chemistry, Government College University Faisalabad, Faisalabad, 38000, Pakistan
[5]Department of Chemistry, GLA University, Mathura, Uttar Pradesh, India
[6]Institute of Biodiversity and Ecosystem Research, Bulgarian Academy of Sciences, 1113 Sofia, Bulgaria
*Correspondence: e.mateev@pharmfac.mu-sofia.bg; mkondeva@pharmfac.mu-sofia.bg

Table of content:

1. Fragments used in the generation of the pyrrole-based database
2. MM/GBSA Ranks of the active MAO-B inhibitors included in the observed dataset, together with the corresponding Specificity, 1-Specificity and Sensitivity.
3. MM/GBSA ranks of the active AChE inhibitors included in the observed dataset, together with the corresponding Specificity, 1-Specificity and Sensitivity.
4. RMSD values of the top ranked 100 solutions with MM/GBSA of the applied MAO-B database. The rejected solutions with RMSD over 2 angstroms were highlighted
5. RMSD values of the top ranked 100 solutions with MM/GBSA of the applied AChE database. The rejected solutions with RMSD over 2 angstrioms were highlighted.
6. Active conformations of EM-DC-21 to EM-DC-27 in MAO-B (PDB:2V5Z)
7. LC/MS, ^{1}H-NMR, and IR spectra of the lead compounds **EM-DC-19** and **EM-DC-27**

8. Active conformations of EM-DC-19 in MAO-B (panels A and B) and in AChE (panels C and D), and of EM-DC-27 in MAO-B (panels E and F) and in AChE (panels G and H).

Supplementary Table S1. Fragments used in the generation of the pyrrole-based database.

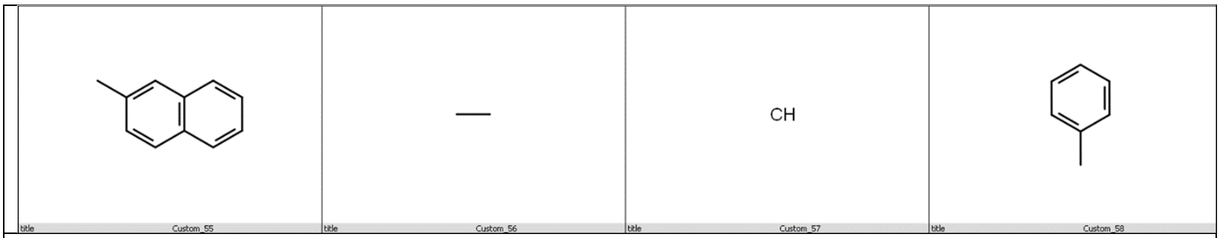

A

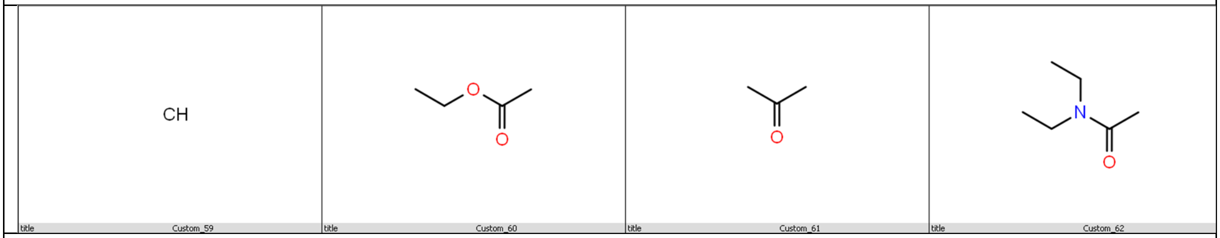

B

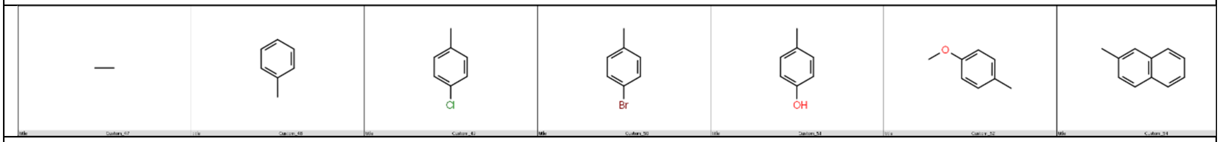

C

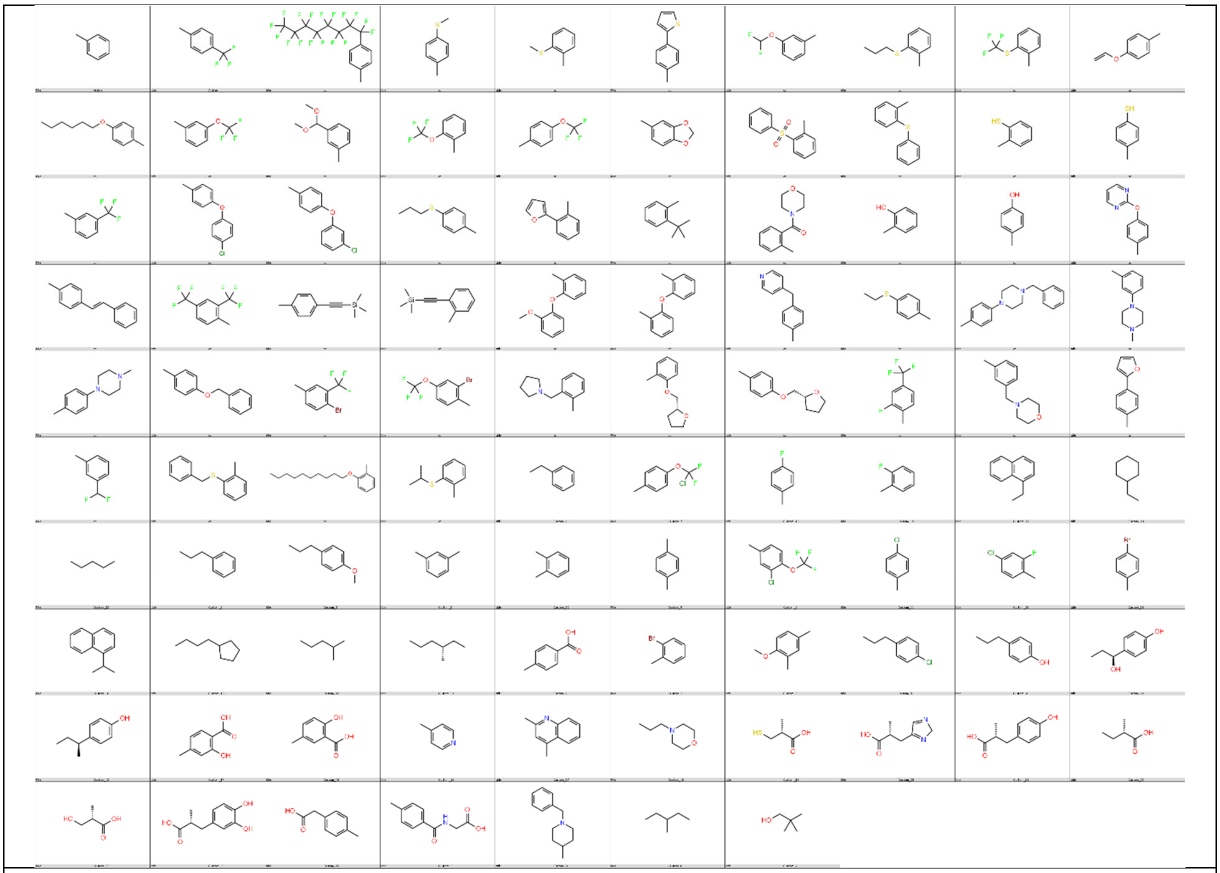

D

(A)- fragments placed in second position of the pyrrole; (B) - fragments placed in third position of the pyrrole; (C) fragments placed in fifth position of the pyrrole; (D) - fragments placed in first position of the pyrrole

Supplementary Table S2. MM/GBSA Ranks of the active MAO-B inhibitors included in the observed dataset, together with the corresponding Specificity, 1-Specificity and Sensitivity.

Title	Rank	Specificity	1-Specificity	Sensitivity
CHEMBL243733	1	1	0	0.008196721
CHEMBL278669	3	0.999845417	0.000154583	0.016393443
CHEMBL333304	20	0.997372082	0.002627918	0.024590164
CHEMBL522271	22	0.997217499	0.002782501	0.032786885
CHEMBL1213972	28	0.996444582	0.003555418	0.040983607
CHEMBL597373	29	0.996444582	0.003555418	0.049180328
CHEMBL598394	34	0.995826248	0.004173752	0.057377049
CHEMBL595	35	0.995826248	0.004173752	0.06557377
CHEMBL127983	37	0.995671665	0.004328335	0.073770492
CHEMBL7002	39	0.995517081	0.004482919	0.081967213
CHEMBL17092	49	0.994125831	0.005874169	0.090163934
CHEMBL18101	56	0.99319833	0.00680167	0.098360656
CHEMBL17078	62	0.992425414	0.007574586	0.106557377
CHEMBL26138	86	0.988869995	0.011130005	0.114754098

CHEMBL16530	91	0.988251662	0.011748338	0.12295082
CHEMBL276076	99	0.987169578	0.012830422	0.131147541
CHEMBL450239	111	0.985469161	0.014530839	0.139344262
CHEMBL348961	117	0.984696244	0.015303756	0.147540984
CHEMBL55674	140	0.981295409	0.018704591	0.155737705
CHEMBL434261	163	0.977894574	0.022105426	0.163934426
CHEMBL459336	202	0.972020405	0.027979595	0.172131148
CHEMBL466872	204	0.971865822	0.028134178	0.180327869
CHEMBL209027	221	0.969392487	0.030607513	0.18852459
CHEMBL470226	223	0.969237904	0.030762096	0.196721311
CHEMBL145330	241	0.966609986	0.033390014	0.204918033
CHEMBL161907	250	0.965373319	0.034626681	0.213114754
CHEMBL488075	284	0.960272067	0.039727933	0.221311475
CHEMBL18317	288	0.959808317	0.040191683	0.229508197
CHEMBL469350	289	0.959808317	0.040191683	0.237704918
CHEMBL274513	318	0.955479981	0.044520019	0.245901639
CHEMBL502021	328	0.954088731	0.045911269	0.254098361
CHEMBL513549	341	0.95223373	0.04776627	0.262295082
CHEMBL406861	362	0.949142062	0.050857938	0.270491803
CHEMBL414637	365	0.948832895	0.051167105	0.278688525
CHEMBL487235	375	0.947441645	0.052558355	0.286885246
CHEMBL589765	386	0.945895811	0.054104189	0.295081967
CHEMBL488074	446	0.93677539	0.06322461	0.303278689
CHEMBL18327	477	0.932137888	0.067862112	0.31147541
CHEMBL19004	479	0.931983305	0.068016695	0.319672131
CHEMBL54762	488	0.930746638	0.069253362	0.327868852
CHEMBL240143	524	0.925336219	0.074663781	0.336065574
CHEMBL16623	539	0.923172051	0.076827949	0.344262295
CHEMBL1213911	551	0.921471634	0.078528366	0.352459016
CHEMBL8145	585	0.916370382	0.083629618	0.360655738
CHEMBL17079	611	0.912505797	0.087494203	0.368852459
CHEMBL570731	619	0.911423713	0.088576287	0.37704918
CHEMBL276915	649	0.906940795	0.093059205	0.385245902
CHEMBL462097	699	0.899366208	0.100633792	0.393442623
CHEMBL457235	726	0.89534704	0.10465296	0.401639344
CHEMBL462054	751	0.891637038	0.108362962	0.409836066
CHEMBL472080	785	0.886535786	0.113464214	0.418032787
CHEMBL394942	792	0.885608286	0.114391714	0.426229508
CHEMBL597372	820	0.881434534	0.118565466	0.43442623
CHEMBL294811	838	0.878806616	0.121193384	0.442622951
CHEMBL464798	861	0.875405781	0.124594219	0.450819672
CHEMBL503807	871	0.874014531	0.125985469	0.459016393
CHEMBL1213718	919	0.866749111	0.133250889	0.467213115
CHEMBL464799	1066	0.844179935	0.155820065	0.475409836
CHEMBL151125	1086	0.841242851	0.158757149	0.483606557
CHEMBL198810	1142	0.832740764	0.167259236	0.491803279
CHEMBL607571	1180	0.827021178	0.172978822	0.5
CHEMBL18832	1240	0.817900757	0.182099243	0.508196721
CHEMBL598393	1288	0.810635338	0.189364662	0.516393443
CHEMBL1213841	1459	0.78435616	0.21564384	0.524590164
CHEMBL325841	1502	0.777863657	0.222136343	0.532786885
CHEMBL1213777	1508	0.77709074	0.22290926	0.540983607
CHEMBL447043	1516	0.776008657	0.223991343	0.549180328
CHEMBL52887	1518	0.775854073	0.224145927	0.557377049
CHEMBL583128	1603	0.762869068	0.237130932	0.56557377
CHEMBL45069	1738	0.742154893	0.257845107	0.573770492
CHEMBL389501	1789	0.734425723	0.265574277	0.581967213

CHEMBL350093	1826	0.72886072	0.27113928	0.590163934
CHEMBL258420	1835	0.727624053	0.272375947	0.598360656
CHEMBL16541	1836	0.727624053	0.272375947	0.606557377
CHEMBL601205	1849	0.725769052	0.274230948	0.614754098
CHEMBL387736	2186	0.673829031	0.326170969	0.62295082
CHEMBL544509	2326	0.652341938	0.347658062	0.631147541
CHEMBL130217	2477	0.629154429	0.370845571	0.639344262
CHEMBL489720	2603	0.609831504	0.390168496	0.647540984
CHEMBL221333	2647	0.603184418	0.396815582	0.655737705
CHEMBL1213968	2694	0.596073582	0.403926418	0.663934426
CHEMBL274029	2696	0.595918998	0.404081002	0.672131148
CHEMBL573301	2704	0.594836915	0.405163085	0.680327869
CHEMBL66979	2719	0.592672747	0.407327253	0.68852459
CHEMBL174289	2749	0.588189828	0.411810172	0.696721311
CHEMBL222368	2784	0.582933993	0.417066007	0.704918033
CHEMBL1213915	2850	0.572886072	0.427113928	0.713114754
CHEMBL267399	3033	0.544751894	0.455248106	0.721311475
CHEMBL425113	3154	0.526201886	0.473798114	0.729508197
CHEMBL18153	3169	0.524037718	0.475962282	0.737704918
CHEMBL79928	3282	0.506724378	0.493275622	0.745901639
CHEMBL295093	3302	0.503787293	0.496212707	0.754098361
CHEMBL298006	3501	0.47317978	0.52682022	0.762295082
CHEMBL450824	3545	0.466532694	0.533467306	0.770491803
CHEMBL389728	3602	0.457876024	0.542123976	0.778688525
CHEMBL269074	3648	0.450919771	0.549080229	0.786885246
CHEMBL6857	3665	0.448446437	0.551553563	0.795081967
CHEMBL224191	3705	0.442417684	0.557582316	0.803278689
CHEMBL269073	3764	0.433451847	0.566548153	0.81147541
CHEMBL455989	3872	0.416911424	0.583088576	0.819672131
CHEMBL39537	3877	0.41629309	0.58370691	0.827868852
CHEMBL607864	3919	0.409955171	0.590044829	0.836065574
CHEMBL552680	3996	0.398206833	0.601793167	0.844262295
CHEMBL31059	4085	0.384603494	0.615396506	0.852459016
CHEMBL596453	4176	0.370690988	0.629309012	0.860655738
CHEMBL315361	4433	0.331117638	0.668882362	0.868852459
CHEMBL374335	4638	0.299582625	0.700417375	0.87704918
CHEMBL592976	4657	0.296800124	0.703199876	0.885245902
CHEMBL14210	4686	0.292471789	0.707528211	0.893442623
CHEMBL269538	4728	0.286133869	0.713866131	0.901639344
CHEMBL10316	4890	0.261245942	0.738754058	0.909836066
CHEMBL404505	5047	0.237130932	0.762869068	0.918032787
CHEMBL266221	5198	0.213943422	0.786056578	0.926229508
CHEMBL275970	5499	0.167568403	0.832431597	0.93442623
CHEMBL363555	5537	0.161848817	0.838151183	0.942622951
CHEMBL92401	5764	0.12691297	0.87308703	0.950819672
CHEMBL171809	6135	0.069717112	0.930282888	0.959016393
CHEMBL441306	6457	0.020095842	0.979904158	0.967213115
CHEMBL14640	6468	0.018550008	0.981449992	0.975409836
CHEMBL57286	6541	0.007420003	0.992579997	0.983606557

Supplementary Table S3. MM/GBSA ranks of the active AChE inhibitors included in the observed dataset, together with the corresponding Specificity, 1-Specificity and Sensitivity.

Title	Rank	Specificity	1-Specificity	Sensitivity	%Screen	%Actives Found

CHEMBL86863	3	0.999924	7.58E-05	0.002208	0.011175	0.220751
CHEMBL610242	8	0.999773	0.000227	0.004415	0.0298	0.441501
CHEMBL73160	11	0.999697	0.000303	0.006623	0.040974	0.662252
CHEMBL521935	12	0.999697	0.000303	0.00883	0.044699	0.883002
CHEMBL362752	13	0.999697	0.000303	0.011038	0.048424	1.103753
CHEMBL609440	16	0.999621	0.000379	0.013245	0.059599	1.324503
CHEMBL87649	20	0.999507	0.000493	0.015453	0.074499	1.545254
CHEMBL87849	22	0.99947	0.00053	0.01766	0.081949	1.766004
CHEMBL187818	31	0.999166	0.000834	0.019868	0.115473	1.986755
CHEMBL76014	33	0.999129	0.000871	0.022075	0.122923	2.207506
CHEMBL365904	36	0.999053	0.000947	0.024283	0.134098	2.428256
CHEMBL212817	39	0.998977	0.001023	0.02649	0.145273	2.649007
CHEMBL474268	47	0.998712	0.001288	0.028698	0.175073	2.869757
CHEMBL225327	50	0.998636	0.001364	0.030905	0.186247	3.090508
CHEMBL502725	59	0.998333	0.001667	0.033113	0.219772	3.311258
CHEMBL189122	60	0.998333	0.001667	0.03532	0.223497	3.532009
CHEMBL82810	73	0.997878	0.002122	0.037528	0.271921	3.752759
CHEMBL87441	92	0.997196	0.002804	0.039735	0.342695	3.97351
CHEMBL1223029	95	0.99712	0.00288	0.041943	0.35387	4.19426
CHEMBL50031	97	0.997083	0.002917	0.04415	0.36132	4.415011
CHEMBL15056	112	0.996552	0.003448	0.046358	0.417194	4.635762
CHEMBL225077	117	0.996401	0.003599	0.048565	0.435819	4.856512
CHEMBL386423	133	0.995832	0.004168	0.050773	0.495418	5.077263
CHEMBL430886	162	0.994771	0.005229	0.05298	0.603442	5.298013
CHEMBL74827	172	0.99443	0.00557	0.055188	0.640691	5.518764
CHEMBL449991	204	0.993256	0.006744	0.057395	0.75989	5.739514
CHEMBL514111	250	0.991551	0.008449	0.059603	0.931237	5.960265
CHEMBL597192	252	0.991513	0.008487	0.06181	0.938687	6.181015
CHEMBL486901	296	0.989884	0.010116	0.064018	1.102585	6.401766
CHEMBL1202835	304	0.989618	0.010382	0.066225	1.132385	6.622517
CHEMBL214881	322	0.988974	0.011026	0.068433	1.199434	6.843267
CHEMBL1088722	324	0.988936	0.011064	0.07064	1.206884	7.064018
CHEMBL518384	344	0.988217	0.011783	0.072848	1.281383	7.284768
CHEMBL223443	364	0.987497	0.012503	0.075055	1.355882	7.505519
CHEMBL456130	382	0.986853	0.013147	0.077263	1.422931	7.726269
CHEMBL1202859	390	0.986587	0.013413	0.07947	1.45273	7.94702
CHEMBL475887	395	0.986436	0.013564	0.081678	1.471355	8.16777
CHEMBL1084256	403	0.986171	0.013829	0.083885	1.501155	8.388521
CHEMBL1084369	411	0.985905	0.014095	0.086093	1.530954	8.609272
CHEMBL1084545	431	0.985185	0.014815	0.0883	1.605453	8.830022
CHEMBL456967	494	0.982836	0.017164	0.090508	1.840125	9.050773
CHEMBL74607	497	0.982761	0.017239	0.092715	1.8513	9.271523
CHEMBL199585	498	0.982761	0.017239	0.094923	1.855025	9.492274
CHEMBL1087458	517	0.982079	0.017921	0.09713	1.925799	9.713024
CHEMBL1087968	521	0.981965	0.018035	0.099338	1.940699	9.933775
CHEMBL1202881	524	0.981889	0.018111	0.101545	1.951874	10.15453
CHEMBL108313	584	0.979654	0.020346	0.103753	2.175371	10.37528
CHEMBL490866	599	0.979123	0.020877	0.10596	2.231245	10.59603
CHEMBL180483	610	0.978744	0.021256	0.108168	2.272219	10.81678
CHEMBL484306	642	0.97757	0.02243	0.110375	2.391418	11.03753
CHEMBL76658	646	0.977456	0.022544	0.112583	2.406318	11.25828
CHEMBL1086007	653	0.977229	0.022771	0.11479	2.432392	11.47903
CHEMBL1202836	672	0.976547	0.023453	0.116998	2.503166	11.69978
CHEMBL424833	673	0.976547	0.023453	0.119205	2.506891	11.92053
CHEMBL1083982	676	0.976471	0.023529	0.121413	2.518066	12.14128
CHEMBL1202878	684	0.976206	0.023794	0.12362	2.547866	12.36203
CHEMBL465028	696	0.975789	0.024211	0.125828	2.592565	12.58278
CHEMBL1202877	705	0.975486	0.024514	0.128035	2.62609	12.80353
CHEMBL255304	707	0.975448	0.024552	0.130243	2.633539	13.02428
CHEMBL517153	741	0.974198	0.025802	0.13245	2.760188	13.24503
CHEMBL1085939	742	0.974198	0.025802	0.134658	2.763913	13.46578
CHEMBL225021	766	0.973326	0.026674	0.136865	2.853311	13.68653
CHEMBL389192	772	0.973137	0.026863	0.139073	2.875661	13.90728
CHEMBL213850	816	0.971508	0.028492	0.14128	3.039559	14.12804
CHEMBL388615	836	0.970788	0.029212	0.143488	3.114058	14.34879
CHEMBL329231	844	0.970522	0.029478	0.145695	3.143858	14.56954
CHEMBL544389	864	0.969803	0.030197	0.147903	3.218357	14.79029
CHEMBL1082845	867	0.969727	0.030273	0.15011	3.229531	15.01104
CHEMBL74747	870	0.969651	0.030349	0.152318	3.240706	15.23179
CHEMBL129753	872	0.969613	0.030387	0.154525	3.248156	15.45254
CHEMBL593998	881	0.96931	0.03069	0.156733	3.281681	15.67329
CHEMBL1084096	925	0.967681	0.032319	0.15894	3.445578	15.89404
CHEMBL108696	945	0.966961	0.033039	0.161148	3.520077	16.11479
CHEMBL319003	950	0.966809	0.033191	0.163355	3.538702	16.33554
CHEMBL545317	956	0.96662	0.03338	0.165563	3.561052	16.55629

CHEMBL110268	957	0.96662	0.03338	0.16777	3.564777	16.77704
CHEMBL141042	974	0.966014	0.033986	0.169978	3.628101	16.99779
CHEMBL447544	979	0.965862	0.034138	0.172185	3.646726	17.21854
CHEMBL445454	1006	0.964877	0.035123	0.174393	3.747299	17.43929
CHEMBL540607	1025	0.964195	0.035805	0.1766	3.818073	17.66004
CHEMBL464427	1037	0.963778	0.036222	0.178808	3.862773	17.88079
CHEMBL219262	1057	0.963058	0.036942	0.181015	3.937272	18.10155
CHEMBL484705	1084	0.962073	0.037927	0.183223	4.037845	18.3223
CHEMBL194823	1085	0.962073	0.037927	0.18543	4.04157	18.54305
CHEMBL140999	1087	0.962035	0.037965	0.187638	4.04902	18.7638
CHEMBL456767	1119	0.960861	0.039139	0.189845	4.168219	18.98455
CHEMBL124156	1146	0.959876	0.040124	0.192053	4.268792	19.2053
CHEMBL238230	1151	0.959724	0.040276	0.19426	4.287417	19.42605
CHEMBL520046	1168	0.959118	0.040882	0.196468	4.350741	19.6468
CHEMBL489454	1176	0.958853	0.041147	0.198675	4.380541	19.86755
CHEMBL538296	1184	0.958588	0.041412	0.200883	4.41034	20.0883
CHEMBL75985	1197	0.958133	0.041867	0.203091	4.458765	20.30905
CHEMBL1082434	1207	0.957792	0.042208	0.205298	4.496014	20.5298
CHEMBL342413	1208	0.957792	0.042208	0.207506	4.499739	20.75055
CHEMBL544159	1283	0.954988	0.045012	0.209713	4.77911	20.9713
CHEMBL185302	1285	0.95495	0.04505	0.211921	4.78656	21.19205
CHEMBL522286	1288	0.954874	0.045126	0.214128	4.797735	21.4128
CHEMBL209382	1319	0.953738	0.046262	0.216336	4.913209	21.63355
CHEMBL475162	1327	0.953473	0.046527	0.218543	4.943008	21.8543
CHEMBL93123	1349	0.952677	0.047323	0.220751	5.024957	22.07506
CHEMBL540833	1363	0.952184	0.047816	0.222958	5.077106	22.29581
CHEMBL590059	1374	0.951805	0.048195	0.225166	5.118081	22.51656
CHEMBL313271	1385	0.951427	0.048573	0.227373	5.159055	22.73731
CHEMBL340625	1400	0.950896	0.049104	0.229581	5.21493	22.95806
CHEMBL87823	1417	0.95029	0.04971	0.231788	5.278254	23.17881
CHEMBL195067	1455	0.948888	0.051112	0.233996	5.419802	23.39956
CHEMBL216002	1492	0.947524	0.052476	0.236203	5.557625	23.62031
CHEMBL179732	1508	0.946956	0.053044	0.238411	5.617224	23.84106
CHEMBL502	1531	0.946122	0.053878	0.240618	5.702898	24.06181
CHEMBL141276	1552	0.945364	0.054636	0.242826	5.781122	24.28256
CHEMBL597188	1556	0.945251	0.054749	0.245033	5.796022	24.50331
CHEMBL595616	1571	0.94472	0.05528	0.247241	5.851896	24.72406
CHEMBL515550	1599	0.943697	0.056303	0.249448	5.956195	24.94481
CHEMBL225567	1611	0.94328	0.05672	0.251656	6.000894	25.16556
CHEMBL426441	1655	0.941651	0.058349	0.253863	6.164792	25.38631
CHEMBL195418	1663	0.941386	0.058614	0.256071	6.194591	25.60706
CHEMBL544823	1748	0.938203	0.061797	0.258278	6.511212	25.82781
CHEMBL555107	1756	0.937938	0.062062	0.260486	6.541012	26.04857
CHEMBL337999	1757	0.937938	0.062062	0.262693	6.544737	26.26932
CHEMBL65018	1764	0.937711	0.062289	0.264901	6.570811	26.49007
CHEMBL192682	1827	0.935362	0.064638	0.267108	6.805483	26.71082
CHEMBL1202834	1832	0.93521	0.06479	0.269316	6.824108	26.93157
CHEMBL140106	1844	0.934793	0.065207	0.271523	6.868807	27.15232
CHEMBL1086246	1848	0.93468	0.06532	0.273731	6.883707	27.37307
CHEMBL567472	1859	0.934301	0.065699	0.275938	6.924682	27.59382
CHEMBL73637	1873	0.933808	0.066192	0.278146	6.976831	27.81457
CHEMBL129108	1874	0.933808	0.066192	0.280353	6.980556	28.03532
CHEMBL77374	1921	0.932065	0.067935	0.282561	7.155628	28.25607
CHEMBL175555	1946	0.931156	0.068844	0.284768	7.248752	28.47682
CHEMBL87344	2007	0.928883	0.071117	0.286976	7.475974	28.69757
CHEMBL299313	2047	0.927405	0.072595	0.289183	7.624972	28.91832
CHEMBL488590	2088	0.925889	0.074111	0.291391	7.777695	29.13907
CHEMBL188714	2101	0.925435	0.074565	0.293598	7.826119	29.35982
CHEMBL75305	2109	0.92517	0.07483	0.295806	7.855919	29.58057
CHEMBL95020	2120	0.924791	0.075209	0.298013	7.896893	29.80132
CHEMBL312310	2166	0.923086	0.076914	0.300221	8.068241	30.02208
CHEMBL1085940	2203	0.921722	0.078278	0.302428	8.206064	30.24283
CHEMBL100819	2237	0.920471	0.079529	0.304636	8.332713	30.46358
CHEMBL1083488	2239	0.920433	0.079567	0.306843	8.340162	30.68433
CHEMBL338755	2244	0.920282	0.079718	0.309051	8.358787	30.90508
CHEMBL1083065	2261	0.919676	0.080324	0.311258	8.422111	31.12583
CHEMBL476438	2264	0.9196	0.0804	0.313466	8.433286	31.34658
CHEMBL1164261	2279	0.919069	0.080931	0.315673	8.48916	31.56733
CHEMBL1085932	2287	0.918804	0.081196	0.317881	8.51896	31.78808
CHEMBL87273	2298	0.918425	0.081575	0.320088	8.559934	32.00883
CHEMBL490264	2322	0.917554	0.082446	0.322296	8.649333	32.22958
CHEMBL477140	2364	0.916	0.084	0.324503	8.805781	32.45033
CHEMBL482512	2365	0.916	0.084	0.326711	8.809506	32.67108
CHEMBL544592	2378	0.915546	0.084454	0.328918	8.85793	32.89183
CHEMBL490660	2400	0.91475	0.08525	0.331126	8.939879	33.11258

CHEMBL1083069	2448	0.912969	0.087031	0.333333	9.118677	33.33333
CHEMBL467760	2485	0.911605	0.088395	0.335541	9.2565	33.55408
CHEMBL106739	2554	0.909029	0.090971	0.337748	9.513522	33.77483
CHEMBL235014	2556	0.908991	0.091009	0.339956	9.520971	33.99558
CHEMBL599650	2576	0.908271	0.091729	0.342163	9.59547	34.21634
CHEMBL328102	2645	0.905695	0.094305	0.344371	9.852492	34.43709
CHEMBL76173	2690	0.904028	0.095972	0.346578	10.02011	34.65784
CHEMBL553263	2715	0.903118	0.096882	0.348786	10.11324	34.87859
CHEMBL127527	2731	0.90255	0.09745	0.350993	10.17284	35.09934
CHEMBL273051	2737	0.90236	0.09764	0.353201	10.19519	35.32009
CHEMBL424707	2756	0.901678	0.098322	0.355408	10.26596	35.54084
CHEMBL55968	2826	0.899064	0.100936	0.357616	10.52671	35.76159
CHEMBL589496	2829	0.898988	0.101012	0.359823	10.53788	35.98234
CHEMBL384886	2879	0.897132	0.102868	0.362031	10.72413	36.20309
CHEMBL557181	2906	0.896147	0.103853	0.364238	10.8247	36.42384
CHEMBL514370	2909	0.896071	0.103929	0.366446	10.83588	36.64459
CHEMBL1083374	2922	0.895616	0.104384	0.368653	10.8843	36.86534
CHEMBL515813	3009	0.892358	0.107642	0.370861	11.20837	37.08609
CHEMBL219264	3055	0.890653	0.109347	0.373068	11.37972	37.30684
CHEMBL475688	3060	0.890501	0.109499	0.375276	11.39835	37.52759
CHEMBL286690	3061	0.890501	0.109499	0.377483	11.40207	37.74834
CHEMBL329012	3085	0.88963	0.11037	0.379691	11.49147	37.96909
CHEMBL263322	3095	0.889289	0.110711	0.381898	11.52872	38.18985
CHEMBL1080691	3121	0.888342	0.111658	0.384106	11.62557	38.4106
CHEMBL1082738	3159	0.88694	0.11306	0.386313	11.76712	38.63135
CHEMBL1203534	3198	0.8855	0.1145	0.388521	11.91239	38.8521
CHEMBL1082849	3202	0.885386	0.114614	0.390728	11.92729	39.07285
CHEMBL154211	3269	0.882886	0.117114	0.392936	12.17686	39.2936
CHEMBL1204299	3272	0.88281	0.11719	0.395143	12.18804	39.51435
CHEMBL244230	3276	0.882696	0.117304	0.397351	12.20294	39.7351
CHEMBL126354	3322	0.880991	0.119009	0.399558	12.37428	39.95585
CHEMBL425739	3348	0.880044	0.119956	0.401766	12.47113	40.1766
CHEMBL361766	3375	0.879059	0.120941	0.403974	12.57171	40.39735
CHEMBL87320	3422	0.877316	0.122684	0.406181	12.74678	40.6181
CHEMBL54058	3426	0.877202	0.122798	0.408389	12.76168	40.83885
CHEMBL1202885	3439	0.876748	0.123252	0.410596	12.8101	41.0596
CHEMBL397271	3440	0.876748	0.123252	0.412804	12.81383	41.28035
CHEMBL278963	3497	0.874626	0.125374	0.415011	13.02615	41.5011
CHEMBL367067	3546	0.872807	0.127193	0.417219	13.20867	41.72185
CHEMBL184784	3561	0.872277	0.127723	0.419426	13.26455	41.9426
CHEMBL87014	3612	0.870382	0.129618	0.421634	13.45452	42.16336
CHEMBL219316	3643	0.869246	0.130754	0.423841	13.56999	42.38411
CHEMBL557405	3655	0.868829	0.131171	0.426049	13.61469	42.60486
CHEMBL243271	3714	0.866631	0.133369	0.428256	13.83446	42.82561
CHEMBL406885	3724	0.86629	0.13371	0.430464	13.87171	43.04636
CHEMBL364125	3753	0.865229	0.134771	0.432671	13.97974	43.26711
CHEMBL478666	3755	0.865192	0.134808	0.434879	13.98719	43.48786
CHEMBL612100	3774	0.86451	0.13549	0.437086	14.05796	43.70861
CHEMBL216159	3777	0.864434	0.135566	0.439294	14.06914	43.92936
CHEMBL108434	3798	0.863676	0.136324	0.441501	14.14736	44.15011
CHEMBL431519	3804	0.863487	0.136513	0.443709	14.16971	44.37086
CHEMBL514534	3810	0.863297	0.136703	0.445916	14.19206	44.59161
CHEMBL61274	4017	0.855492	0.144508	0.448124	14.96312	44.81236
CHEMBL260964	4018	0.855492	0.144508	0.450331	14.96685	45.03311
CHEMBL544126	4019	0.855492	0.144508	0.452539	14.97057	45.25386
CHEMBL345847	4046	0.854507	0.145493	0.454746	15.07115	45.47461
CHEMBL110627	4047	0.854507	0.145493	0.456954	15.07487	45.69536
CHEMBL599644	4080	0.853294	0.146706	0.459161	15.19779	45.91611
CHEMBL542467	4082	0.853257	0.146743	0.461369	15.20524	46.13687
CHEMBL555666	4097	0.852726	0.147274	0.463576	15.26112	46.35762
CHEMBL315515	4102	0.852575	0.147425	0.465784	15.27974	46.57837
CHEMBL186144	4118	0.852006	0.147994	0.467991	15.33934	46.79912
CHEMBL153865	4150	0.850832	0.149168	0.470199	15.45854	47.01987
CHEMBL128260	4176	0.849884	0.150116	0.472406	15.55539	47.24062
CHEMBL410915	4189	0.84943	0.15057	0.474614	15.60381	47.46137
CHEMBL131827	4337	0.84386	0.15614	0.476821	16.15511	47.68212
CHEMBL160095	4411	0.841094	0.158906	0.479029	16.43075	47.90287
CHEMBL478706	4446	0.839806	0.160194	0.481236	16.56113	48.12362
CHEMBL599652	4484	0.838404	0.161596	0.483444	16.70267	48.34437
CHEMBL155746	4526	0.836851	0.163149	0.485651	16.85912	48.56512
CHEMBL360832	4537	0.836472	0.163528	0.487859	16.9001	48.78587
CHEMBL255502	4584	0.834729	0.165271	0.490066	17.07517	49.00662
CHEMBL429660	4654	0.832115	0.167885	0.492274	17.33592	49.22737
CHEMBL1085858	4668	0.831622	0.168378	0.494481	17.38807	49.44812
CHEMBL413241	4677	0.831319	0.168681	0.496689	17.42159	49.66887

CHEMBL396158	4706	0.830258	0.169742	0.498896	17.52961	49.88962
CHEMBL433678	4707	0.830258	0.169742	0.501104	17.53334	50.11038
CHEMBL1202865	4744	0.828894	0.171106	0.503311	17.67116	50.33113
CHEMBL475685	4765	0.828136	0.171864	0.505519	17.74939	50.55188
CHEMBL396199	4772	0.827909	0.172091	0.507726	17.77546	50.77263
CHEMBL308962	4794	0.827113	0.172887	0.509934	17.85741	50.99338
CHEMBL145446	4837	0.825522	0.174478	0.512141	18.01758	51.21413
CHEMBL107516	4852	0.824991	0.175009	0.514349	18.07346	51.43488
CHEMBL537908	4853	0.824991	0.175009	0.516556	18.07718	51.65563
CHEMBL589419	4887	0.823741	0.176259	0.518764	18.20383	51.87638
CHEMBL100718	4915	0.822718	0.177282	0.520971	18.30813	52.09713
CHEMBL76470	4924	0.822415	0.177585	0.523179	18.34165	52.31788
CHEMBL124933	4969	0.820748	0.179252	0.525386	18.50928	52.53863
CHEMBL219911	4984	0.820217	0.179783	0.527594	18.56515	52.75938
CHEMBL209392	4987	0.820142	0.179858	0.529801	18.57632	52.98013
CHEMBL481	4990	0.820066	0.179934	0.532009	18.5875	53.20088
CHEMBL1084775	5150	0.814042	0.185958	0.534216	19.18349	53.42163
CHEMBL108632	5182	0.812867	0.187133	0.536424	19.30269	53.64238
CHEMBL445846	5238	0.810783	0.189217	0.538631	19.51129	53.86313
CHEMBL56001	5261	0.80995	0.19005	0.540839	19.59696	54.08389
CHEMBL1202884	5344	0.806843	0.193157	0.543046	19.90613	54.30464
CHEMBL156157	5405	0.804569	0.195431	0.545254	20.13335	54.52539
CHEMBL602861	5414	0.804266	0.195734	0.547461	20.16688	54.74614
CHEMBL224202	5433	0.803584	0.196416	0.549669	20.23765	54.96689
CHEMBL483090	5434	0.803584	0.196416	0.551876	20.24138	55.18764
CHEMBL48643	5448	0.803092	0.196908	0.554084	20.29353	55.40839
CHEMBL243270	5528	0.800099	0.199901	0.556291	20.59152	55.62914
CHEMBL140476	5562	0.798848	0.201152	0.558499	20.71817	55.84989
CHEMBL599651	5654	0.7954	0.2046	0.560706	21.06087	56.07064
CHEMBL378404	5676	0.794605	0.205395	0.562914	21.14281	56.29139
CHEMBL1085030	5697	0.793847	0.206153	0.565121	21.22104	56.51214
CHEMBL64011	5711	0.793354	0.206646	0.567329	21.27319	56.73289
CHEMBL610243	5714	0.793279	0.206721	0.569536	21.28436	56.95364
CHEMBL224998	5756	0.791725	0.208275	0.571744	21.44081	57.17439
CHEMBL267591	5764	0.79146	0.20854	0.573951	21.47061	57.39514
CHEMBL609668	5838	0.788694	0.211306	0.576159	21.74626	57.61589
CHEMBL340427	5857	0.788012	0.211988	0.578366	21.81703	57.83664
CHEMBL542543	5878	0.787254	0.212746	0.580574	21.89525	58.0574
CHEMBL473866	5933	0.785208	0.214792	0.582781	22.10013	58.27815
CHEMBL117521	5934	0.785208	0.214792	0.584989	22.10385	58.4989
CHEMBL1085933	5948	0.784716	0.215284	0.587196	22.156	58.71965
CHEMBL1082739	6007	0.782518	0.217482	0.589404	22.37577	58.9404
CHEMBL156874	6017	0.782177	0.217823	0.591611	22.41302	59.16115
CHEMBL519154	6074	0.780055	0.219945	0.593819	22.62534	59.3819
CHEMBL385269	6269	0.772705	0.227295	0.596026	23.35171	59.60265
CHEMBL140228	6290	0.771947	0.228053	0.598234	23.42993	59.8234
CHEMBL155945	6322	0.770773	0.229227	0.600442	23.54913	60.04415
CHEMBL256919	6401	0.767817	0.232183	0.602649	23.8434	60.2649
CHEMBL128471	6587	0.760808	0.239192	0.604857	24.53624	60.48565
CHEMBL1084530	6713	0.756072	0.243928	0.607064	25.00559	60.7064
CHEMBL93241	6733	0.755352	0.244648	0.609272	25.08009	60.92715
CHEMBL1084703	6789	0.753268	0.246732	0.611479	25.28868	61.1479
CHEMBL372491	6889	0.749517	0.250483	0.613687	25.66118	61.36865
CHEMBL307908	6935	0.747812	0.252188	0.615894	25.83253	61.5894
CHEMBL142838	6977	0.746258	0.253742	0.618102	25.98897	61.81015
CHEMBL554252	7033	0.744175	0.255825	0.620309	26.19757	62.03091
CHEMBL306492	7211	0.737468	0.262532	0.622517	26.86061	62.25166
CHEMBL606372	7268	0.735346	0.264654	0.624724	27.07293	62.47241
CHEMBL362049	7274	0.735157	0.264843	0.626932	27.09528	62.69316
CHEMBL403083	7281	0.73493	0.26507	0.629139	27.12136	62.91391
CHEMBL190322	7312	0.733793	0.266207	0.631347	27.23683	63.13466
CHEMBL158252	7387	0.730989	0.269011	0.633554	27.5162	63.35541
CHEMBL1172762	7406	0.730307	0.269693	0.635762	27.58698	63.57616
CHEMBL154972	7458	0.728375	0.271625	0.637969	27.78067	63.79691
CHEMBL1173042	7511	0.726405	0.273595	0.640177	27.9781	64.01766
CHEMBL145713	7589	0.723487	0.276513	0.642384	28.26864	64.23841
CHEMBL542497	7611	0.722692	0.277308	0.644592	28.35059	64.45916
CHEMBL187870	7652	0.721176	0.278824	0.646799	28.50332	64.67991
CHEMBL594229	7693	0.719661	0.280339	0.649007	28.65604	64.90066
CHEMBL464537	7716	0.718827	0.281173	0.651214	28.74171	65.12141
CHEMBL110554	7720	0.718713	0.281287	0.653422	28.75661	65.34216
CHEMBL1173761	7732	0.718297	0.281703	0.655629	28.80131	65.56291
CHEMBL595130	7749	0.71769	0.28231	0.657837	28.86464	65.78366
CHEMBL352500	7795	0.715985	0.284015	0.660044	29.03598	66.00442
CHEMBL158860	7930	0.710908	0.289092	0.662252	29.53885	66.22517

CHEMBL219569	7937	0.710681	0.289319	0.664459	29.56493	66.44592
CHEMBL245470	7980	0.70909	0.29091	0.666667	29.7251	66.66667
CHEMBL1204112	8217	0.700148	0.299852	0.668874	30.60791	66.88742
CHEMBL606348	8235	0.699504	0.300496	0.671082	30.67496	67.10817
CHEMBL1173663	8335	0.695753	0.304247	0.673289	31.04746	67.32892
CHEMBL118752	8343	0.695487	0.304513	0.675497	31.07726	67.54967
CHEMBL92955	8363	0.694768	0.305232	0.677704	31.15175	67.77042
CHEMBL155111	8415	0.692835	0.307165	0.679912	31.34545	67.99117
CHEMBL189907	8445	0.691736	0.308264	0.682119	31.4572	68.21192
CHEMBL1204162	8456	0.691358	0.308642	0.684327	31.49817	68.43267
CHEMBL440983	8506	0.689501	0.310499	0.686534	31.68442	68.65342
CHEMBL388551	8609	0.685636	0.314364	0.688742	32.06809	68.87417
CHEMBL1082846	8648	0.684197	0.315803	0.690949	32.21337	69.09492
CHEMBL128618	8736	0.6809	0.3191	0.693157	32.54116	69.31567
CHEMBL612099	8822	0.67768	0.32232	0.695364	32.86151	69.53642
CHEMBL1087459	8829	0.677452	0.322548	0.697572	32.88758	69.75717
CHEMBL92736	8841	0.677036	0.322964	0.699779	32.93228	69.97792
CHEMBL543655	8888	0.675293	0.324707	0.701987	33.10735	70.19868
CHEMBL370807	8899	0.674914	0.325086	0.704194	33.14833	70.41943
CHEMBL107572	8918	0.674232	0.325768	0.706402	33.2191	70.64018
CHEMBL322392	8959	0.672716	0.327284	0.708609	33.37182	70.86093
CHEMBL321605	9087	0.667904	0.332096	0.710817	33.84862	71.08168
CHEMBL590055	9192	0.663964	0.336036	0.713024	34.23974	71.30243
CHEMBL140990	9282	0.660592	0.339408	0.715232	34.57498	71.52318
CHEMBL284102	9393	0.656424	0.343576	0.717439	34.98845	71.74393
CHEMBL191386	9404	0.656045	0.343955	0.719647	35.02943	71.96468
CHEMBL555075	9618	0.647975	0.352025	0.721854	35.82657	72.18543
CHEMBL427082	9765	0.642443	0.357557	0.724062	36.37413	72.40618
CHEMBL1084262	9909	0.637025	0.362975	0.726269	36.91053	72.62693
CHEMBL1085031	9933	0.636154	0.363846	0.728477	36.99993	72.84768
CHEMBL158637	10052	0.631683	0.368317	0.730684	37.44319	73.06843
CHEMBL223549	10053	0.631683	0.368317	0.732892	37.44692	73.28918
CHEMBL1096994	10090	0.630319	0.369681	0.735099	37.58474	73.50993
CHEMBL347667	10187	0.626681	0.373319	0.737307	37.94606	73.73068
CHEMBL597801	10223	0.625355	0.374645	0.739514	38.08016	73.95143
CHEMBL364545	10264	0.62384	0.37616	0.741722	38.23288	74.17219
CHEMBL9751	10295	0.622703	0.377297	0.743929	38.34836	74.39294
CHEMBL91538	10428	0.617702	0.382298	0.746137	38.84378	74.61369
CHEMBL197696	10664	0.608798	0.391202	0.748344	39.72286	74.83444
CHEMBL1172618	10728	0.606411	0.393589	0.750552	39.96126	75.05519
CHEMBL550075	10965	0.597469	0.402531	0.752759	40.84407	75.27594
CHEMBL1084263	11028	0.59512	0.40488	0.754967	41.07875	75.49669
CHEMBL349694	11179	0.589437	0.410563	0.757174	41.64121	75.71744
CHEMBL161779	11229	0.58758	0.41242	0.759382	41.82746	75.93819
CHEMBL211520	11272	0.585989	0.414011	0.761589	41.98763	76.15894
CHEMBL219550	11348	0.583147	0.416853	0.763797	42.27073	76.37969
CHEMBL210453	11436	0.579851	0.420149	0.766004	42.59852	76.60044
CHEMBL539045	11458	0.579055	0.420945	0.768212	42.68047	76.82119
CHEMBL159576	11583	0.574357	0.425643	0.770419	43.14609	77.04194
CHEMBL589978	11717	0.569318	0.430682	0.772627	43.64524	77.26269
CHEMBL392324	11917	0.561778	0.438222	0.774834	44.39023	77.48344
CHEMBL311017	11968	0.559883	0.440117	0.777042	44.5802	77.70419
CHEMBL595129	11993	0.558974	0.441026	0.779249	44.67332	77.92494
CHEMBL406645	12072	0.556019	0.443981	0.781457	44.96759	78.1457
CHEMBL594225	12181	0.551927	0.448073	0.783664	45.37361	78.36645
CHEMBL175487	12212	0.55079	0.44921	0.785872	45.48909	78.5872
CHEMBL220141	12295	0.547683	0.452317	0.788079	45.79826	78.80795
CHEMBL56960	12560	0.53768	0.46232	0.790287	46.78537	79.0287
CHEMBL93936	12616	0.535597	0.464403	0.792494	46.99397	79.24945
CHEMBL83005	12731	0.531277	0.468723	0.794702	47.42233	79.4702
CHEMBL332051	12776	0.52961	0.47039	0.796909	47.58996	79.69095
CHEMBL223277	12869	0.526124	0.473876	0.799117	47.93638	79.9117
CHEMBL596513	12991	0.52154	0.47846	0.801325	48.39082	80.13245
CHEMBL542220	13076	0.518357	0.481643	0.803532	48.70744	80.3532
CHEMBL339995	13138	0.516046	0.483954	0.80574	48.93839	80.57395
CHEMBL1084544	13172	0.514796	0.485204	0.807947	49.06504	80.7947
CHEMBL1080692	13364	0.507559	0.492441	0.810155	49.78023	81.01545
CHEMBL307004	13494	0.502671	0.497329	0.812362	50.26447	81.2362
CHEMBL62664	13572	0.499754	0.500246	0.81457	50.55502	81.45695
CHEMBL554452	13666	0.49623	0.50377	0.816777	50.90516	81.6777
CHEMBL335033	13823	0.490319	0.509681	0.818985	51.48998	81.89845
CHEMBL477141	13848	0.48941	0.51059	0.821192	51.5831	82.11921
CHEMBL225198	14035	0.482363	0.517637	0.8234	52.27967	82.33996
CHEMBL478667	14126	0.478953	0.521047	0.825607	52.61864	82.56071
CHEMBL557155	14136	0.478612	0.521388	0.827815	52.65589	82.78146

CHEMBL1087194	14191	0.476566	0.523434	0.830022	52.86076	83.00221
CHEMBL224335	14201	0.476225	0.523775	0.83223	52.89801	83.22296
CHEMBL44080	14267	0.473762	0.526238	0.834437	53.14386	83.44371
CHEMBL478193	14340	0.471034	0.528966	0.836645	53.41578	83.66446
CHEMBL342564	14349	0.470731	0.529269	0.838852	53.4493	83.88521
CHEMBL257126	14567	0.462509	0.537491	0.84106	54.26134	84.10596
CHEMBL65585	14638	0.459857	0.540143	0.843267	54.52581	84.32671
CHEMBL430679	14706	0.457318	0.542682	0.845475	54.77911	84.54746
CHEMBL42531	14786	0.454325	0.545675	0.847682	55.07711	84.76821
CHEMBL362444	15029	0.445156	0.554844	0.84989	55.98227	84.98896
CHEMBL426015	15114	0.441973	0.558027	0.852097	56.29889	85.20971
CHEMBL336524	15155	0.440458	0.559542	0.854305	56.45161	85.43046
CHEMBL345241	15222	0.437957	0.562043	0.856512	56.70118	85.65121
CHEMBL158946	15223	0.437957	0.562043	0.85872	56.70491	85.87196
CHEMBL1083662	15532	0.426287	0.573713	0.860927	57.85592	86.09272
CHEMBL1083660	15633	0.422498	0.577502	0.863135	58.23214	86.31347
CHEMBL427232	15667	0.421248	0.578752	0.865342	58.35879	86.53422
CHEMBL603622	15756	0.417914	0.582086	0.86755	58.69031	86.75497
CHEMBL158232	15871	0.413595	0.586405	0.869757	59.11868	86.97572
CHEMBL121810	16117	0.404312	0.595688	0.871965	60.03501	87.19647
CHEMBL490338	16200	0.401205	0.598795	0.874172	60.34419	87.41722
CHEMBL159702	16709	0.381957	0.618043	0.87638	62.24018	87.63797
CHEMBL7634	16828	0.377486	0.622514	0.878587	62.68345	87.85872
CHEMBL219063	17015	0.370439	0.629561	0.880795	63.38002	88.07947
CHEMBL130373	17089	0.367673	0.632327	0.883002	63.65567	88.30022
CHEMBL395280	17852	0.338802	0.661198	0.88521	66.4978	88.52097
CHEMBL89354	17903	0.336908	0.663092	0.887417	66.68777	88.74172
CHEMBL473050	17923	0.336188	0.663812	0.889625	66.76227	88.96247
CHEMBL266045	18016	0.332702	0.667298	0.891832	67.10869	89.18322
CHEMBL223714	18147	0.327776	0.672224	0.89404	67.59666	89.40397
CHEMBL148522	18164	0.32717	0.67283	0.896247	67.65999	89.62472
CHEMBL582883	18251	0.323912	0.676088	0.898455	67.98406	89.84547
CHEMBL52722	18536	0.313151	0.686849	0.900662	69.04567	90.06623
CHEMBL422695	18592	0.311067	0.688933	0.90287	69.25427	90.28698
CHEMBL146674	18625	0.309855	0.690145	0.905077	69.37719	90.50773
CHEMBL195241	18664	0.308415	0.691585	0.907285	69.52246	90.72848
CHEMBL322994	18690	0.307468	0.692532	0.909492	69.61931	90.94923
CHEMBL79874	19485	0.277384	0.722616	0.9117	72.58065	91.16998
CHEMBL335158	19595	0.273254	0.726746	0.913907	72.99039	91.39073
CHEMBL134342	19935	0.26041	0.73959	0.916115	74.25687	91.61148
CHEMBL491359	20031	0.256811	0.743189	0.918322	74.61447	91.83223
CHEMBL183813	20040	0.256507	0.743493	0.92053	74.64799	92.05298

Supplemtanry Table S4. RMSD values of the top ranked 100 solutions with MM/GBSA of the applied MAO-B database. The rejected solutions with RMSD over 2 angstroms were highlighted.

Compound	RMSD value	Compound	RMSD value
Chembl243733	1.27	chembl595	10.41
ZINC24499981	3.17	zinc49877041	2.01
Chemblb278669	1.22	chembl127983	0.11
zinc05286877	1.55	chembl522271	0.82
zinc23158928	0.63	zinc55082653	1.55
zinc58201116	0.20	chembl7002	0.58
chembl278669	1.94	zinc05164834	0.53
zinc23420948	0.55	zinc11875385	0.74
zinc39197592	0.20	chembl7002	0.47
zinc66376868	2.41	zinc54640784	9.07
zinc65270237	1.25	zinc49896263	1.64
zinc05001822	10.66	zinc05266525	2.46
zinc31714020	9.75	zinc01331989	0.43
zinc22794727	9.63	zinc57762437	8.96
zinc07398348	0.29	zinc12581649	0.50
zinc40261230	1.80	zinc15748130	1.40
zinc06258323	0.28	chembl17092	1.42
zinc04093918	0.34	zinc11849523	0.52
zinc65999169	0.93	zinc31821236	1.51
ziinc61441513	0.93	zinc38076150	1.65
chembl333304	1.59	zinc12592525	0.91
zinc49120613	2.42	zinc61432849	3.02

chembl522271	0.31	zinc67353304	1.13
zinc40248658	0.76	chembl18101	1.88
zinc44029673	9.88	zinc57836514	0.51
zinc66470773	0.73	zinc55200078	0.47
zinc01086857	3.76	zinc15061577	2.09
zinc63206662	0.97	zinc10845956	0.56
chembl1213972	0.85	zinc64050483	3.70
chembl597373	2.71	chembl17078	0.88
zinc49618793	1.11	zinc00090358	0.49
zinc48088413	2.31	zinc06873712	0.48
zinc49896265	2.70	zinc05264344	1.54
zinc62526653	0.54	zinc00149171	1.34
chembl598394	0.56	ZINC17747651	10.06
ZINC12598874	9.51	CHEMBL276076	0.57
ZINC00401142	1.74	ZINC32912802	9.68
ZINC25222777	0.34	CHEMBL450239	3.00
ZINC62288037	4.59	ZINC53601853	2.99
ZINC08849448	1.19	ZINC07437995	1.80
ZINC59843198	0.39	CHEMBL348961	1.60
ZINC40857693	0.71	ZINC14245507	0.69
CHEMBL26138	0.50	ZINC26462750	9.36
ZINC52695721	0.40	CHEMBL598392	0.68
ZINC46735795	10.09	ZINC39746903	2.40
CHEMBL16530	0.91	CHEMBL55674	1.22
ZINC66623335	3.22	CHEMBL434261	1.94
ZINC39766674	0.41	ZINC37149241	9.04
ZINC61803162	0.64	ZINC62801829	0.68
ZINC19635461	0.65	ZINC04040031	0.57

Supplemtanry Table S5. RMSD values of the top ranked 100 solutions with MM/GBSA of the applied AChE database. The rejected solutions with RMSD over 2 angstrioms were highlighted.

Compound	RMSD value	Compound	RMSD value
ZINC01834221	1.86	CHEMBL609440	0.88
CHEMBL545317	1.68	ZINC39950118	9.64
ZINC63137031	2.10	ZINC07990302	10.23
ZINC63229160	2.37	ZINC63312600	3.43
ZINC63075043	1.64	ZINC63196927	4.42
CHEMBL86863	0.15	CHEMBL544389	1.37
ZINC16588540	2.38	CHEMBL610242	2.86
ZINC64074444	2.16	ZINC63416398	10.83
ZINC63591083	2.26	ZINC52566150	3.73
ZINC39396353	1.01	ZINC39357174	5.11
ZINC39934759	2.06	ZINC52627902	3.73
ZINC64143588	3.12	ZINC39670061	4.56
ZINC64074447	9.57	CHEMBL87649	1.74
ZINC27107376	9.00	ZINC63251843	10.55
ZINC64084301	5.38	ZINC65039340	8.98
ZINC39700649	2.68	ZINC39358163	3.48
CHEMBL141276	1.96	ZINC63625921	9.32
CHEMBL87849	2.97	ZINC64743956	3.20
ZINC64131573	3.18	ZINC36065434	2.76
ZINC09846543	11.55	CHEMBL329231	1.94
ZINC23373892	3.40	ZINC19292065	3.17
ZINC39934681	3.26	ZINC12848725	9.56
CHEMBL140106	2.64	ZINC64427224	2.44
ZINC63312601	1.86	ZINC63289658	2.17
CHEMBL141042	1.75	ZINC63886494	2.47
CHEMBL544389	1.63	ZINC39445329	3.79
CHEMBL110268	2.63	CHEMBL154211	1.13
ZINC39934899	2.63	ZINC39703212	3.57
ZINC65113010	3.02	ZINC63561751	0.41
ZINC64143362	9.30	ZINC02294482	2.55
ZINC39934885	10.30	CHEMBL76470	1.78
ZINC63428685	1.83	ZINC39405106	1.40
ZINC39934922	2.66	CHEMBL1084256	1.94
CHEMBL319003	2.32	ZINC22790041	3.94
ZINC39934864	3.54	CHEMBL362752	1.11
ZINC17305805	9.49	CHEMBL82810	1.15

ZINC39489718	3.40	CHEMBL1203534	2.27
ZINC04785879	3.83	CHEMBL555107	1.13
ZINC63291195	2.79	ZINC64143365	3.26
ZINC64044984	9.97	ZINC38566949	8.58
CHEMBL187818	1.82	ZINC58013864	2.73
ZINC63933933	8.45	CHEMBL593998	1.34
ZINC15108671	2.65	ZINC44975512	12.24
ZINC63847142	9.80	CHEMBL145446	2.17
ZINC63330620	4.66	CHEMBL180483	1.13
CHEMBL1223029	0.16	ZINC63386457	2.31
CHEMBL553263	1.89	ZINC36065313	10.55
CHEMBL1082434	2.18	ZINC63696330	2.55
ZINC26813042	9.22	ZINC63696024	1.97
ZINC63985736	4.81	ZINC44800971	10.29

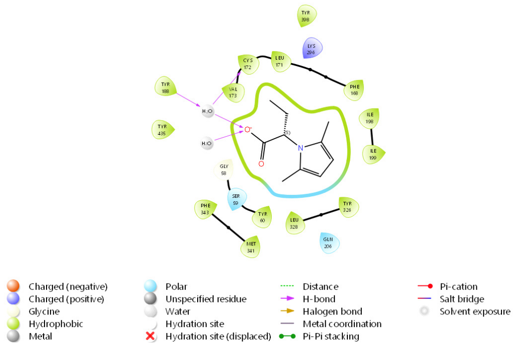

EM-DC-21

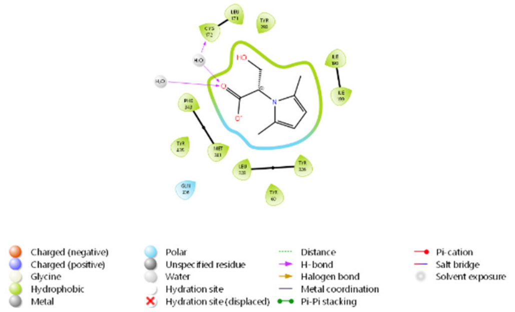

EM-DC-22

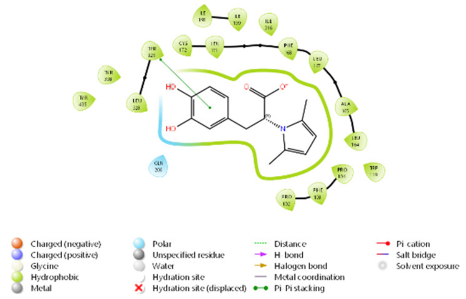

EM-DC-23

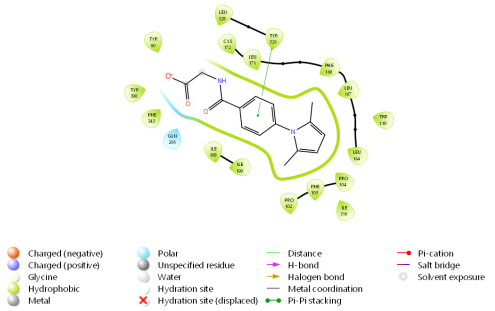

EM-DC-24

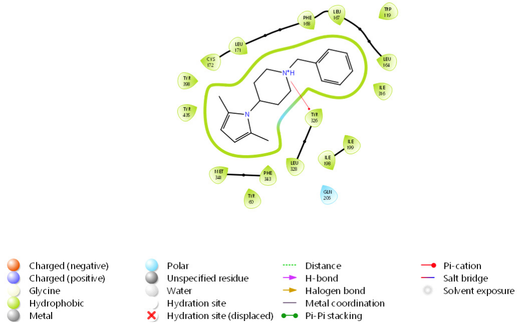

EM-DC-25

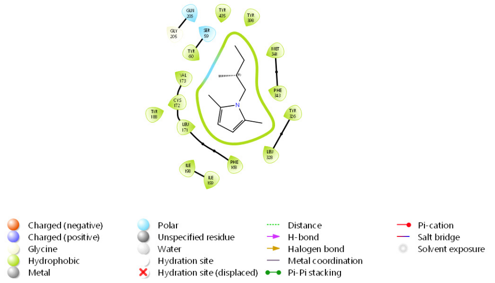

EM-DC-26

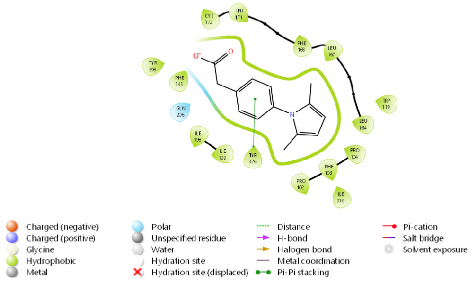

EM-DC-27

Supplementary Figure S1. Active conformations of compounds EM-DC-21 to EM-DC-27 in the MAO-B active site (PDB ID: 2V5Z). The figure shows 2D and 3D docking poses of each compound within the enzyme's binding pocket, highlighting key interactions such as hydrogen bonds and hydrophobic contacts that contribute to binding stability and specificity.

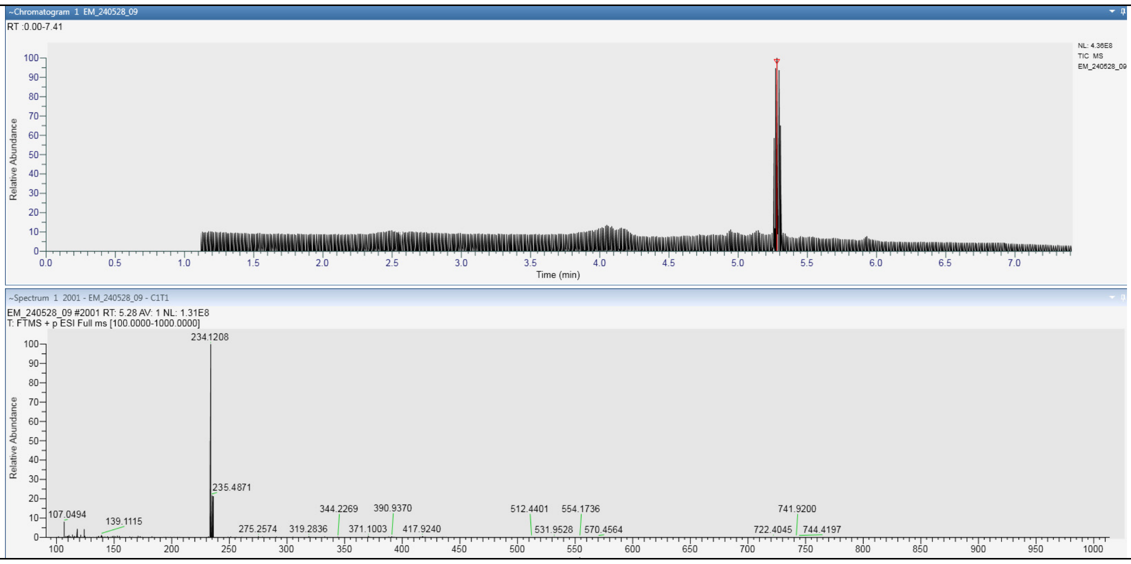

A

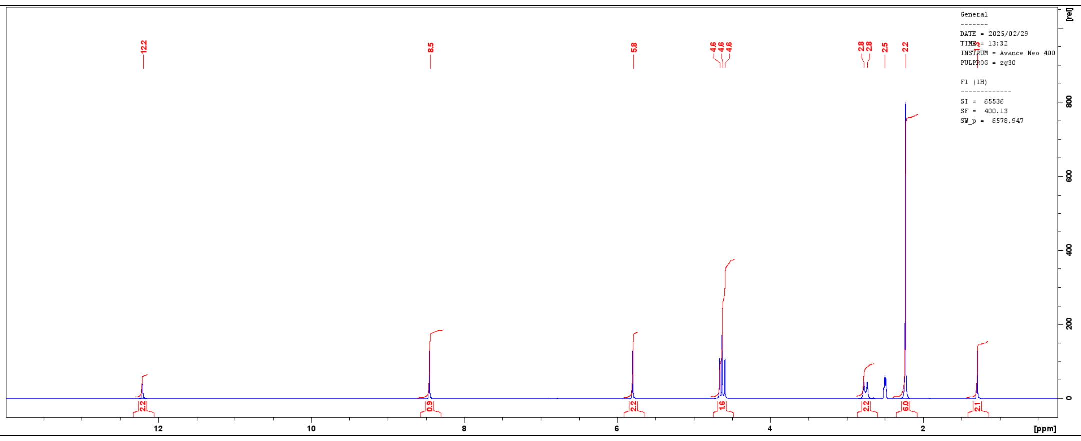

B

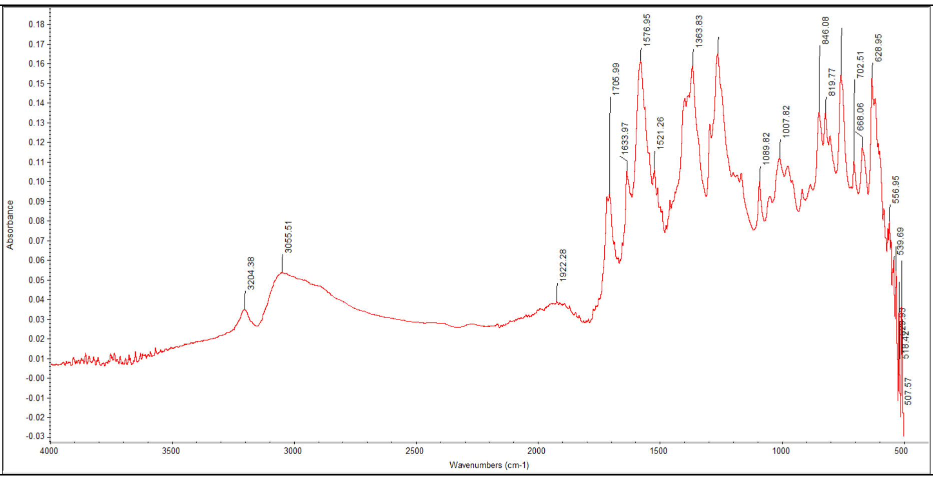
Absorbance
Wavenumbers (cm-1)
3204.38
3055.51
1922.28
1705.99
1633.97
1576.95
1521.26
1363.83
1089.82
1007.82
846.08
819.77
702.51
668.06
628.95
556.95
539.69
507.57

C

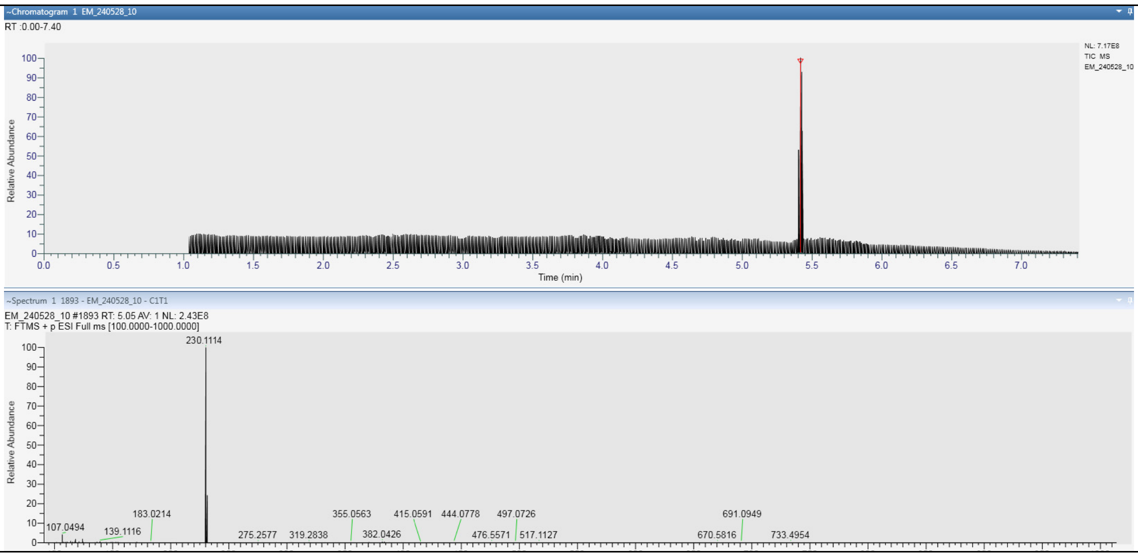
~Chromatogram 1 EM_240528_10
RT :0.00-7.40
NL: 7.17E8
TIC MS
EM_240528_10
Relative Abundance
Time (min)
~Spectrum 1 1893 - EM_240528_10 - C1T1
EM_240528_10 #1893 RT: 5.05 AV: 1 NL: 2.43E8
T: FTMS + p ESI Full ms [100.0000-1000.0000]
Relative Abundance
230.1114
107.0494
139.1116
183.0214
275.2577
319.2838
355.0563
382.0426
415.0591
444.0778
476.5571
497.0726
517.1127
670.5816
691.0949
733.4954

D

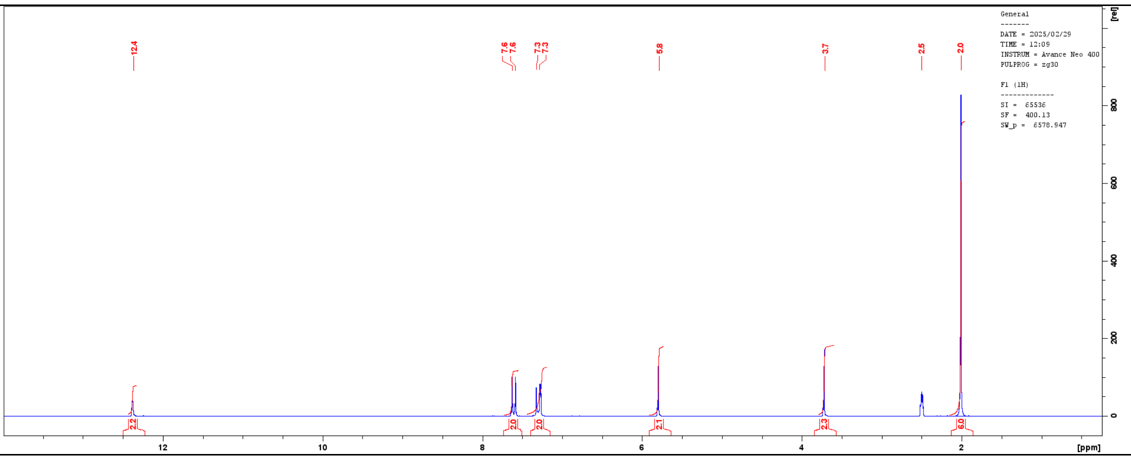
General
DATE = 2025/02/29
TIME = 12:09
INSTRUM = Avance Neo 400
PULPROG = zg30
F1 (1H)
SI = 65536
SF = 400.13
SW_p = 6578.947
12.4
7.6
7.6
7.3
7.3
5.8
3.7
2.5
2.0
[ppm]
[rel]

E

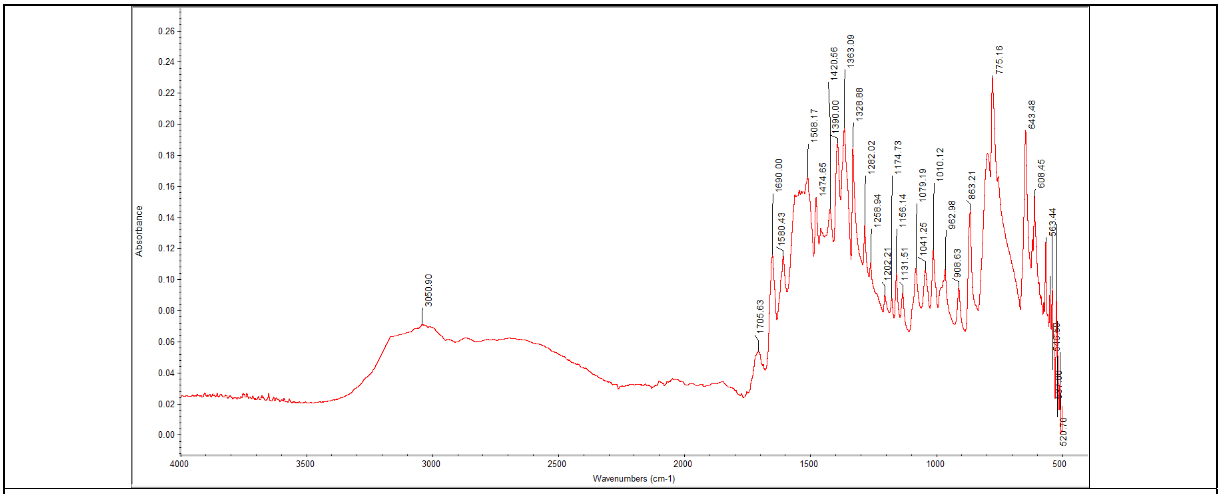

F

Supplementary Figure S2. LC/MS, ^{1}H-NMR, and IR spectra of the lead compounds **EM-DC-19** (panels A, B, C) and **EM-DC-27** (panels D, E, F).

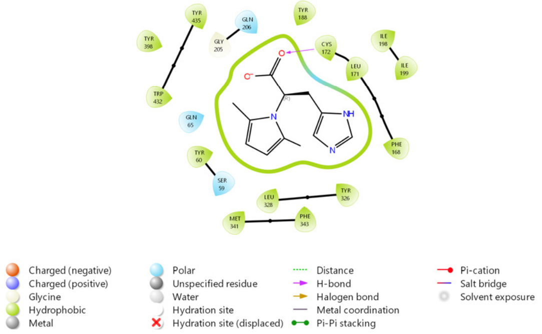

A

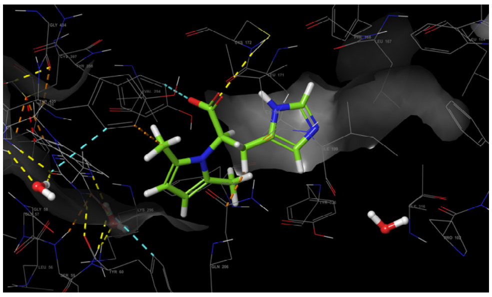

B

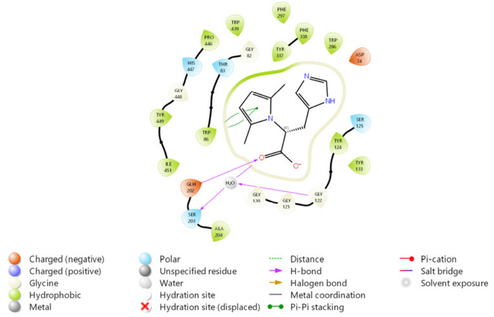

C

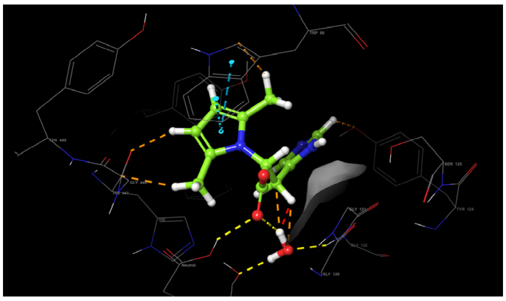

D

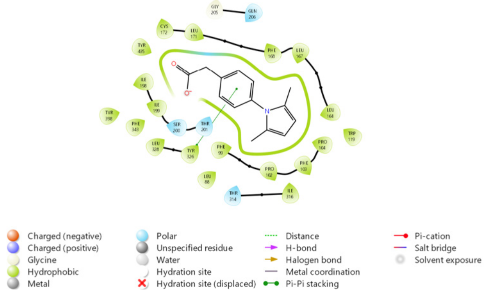

E

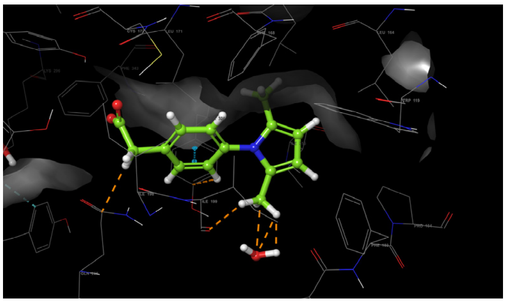

F

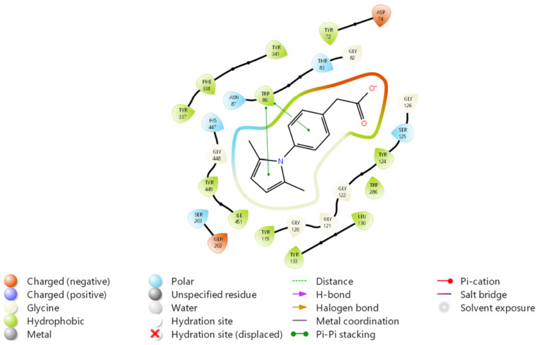

G

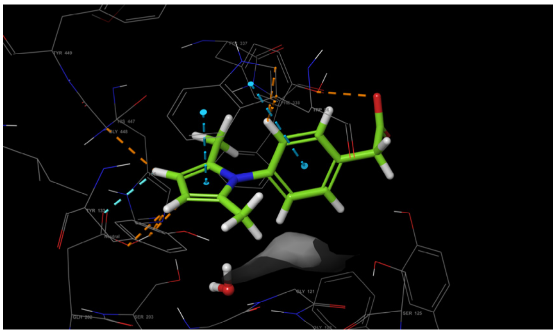

H

Supplementary Figure S3. Active conformations of EM-DC-19 and EM-DC-27 in MAO-B and AChE enzymes. Panels A and B show the docking poses of EM-DC-19 in MAO-B; panels C and D present EM-DC-19 in AChE. Panels E and F illustrate EM-DC-27 in MAO-B, while panels G and H depict EM-DC-27 in AChE. Both 2D and 3D poses demonstrate molecular interactions responsible for enzyme inhibition and selectivity.

Supplementary Table S6. Toxicity Endpoints Predicted by Derek Nexus

5alpha-Reductase inhibition	Mitochondrial dysfunction
Adrenal gland toxicity	Mutagenicity in vivo
alpha-2-mu-Globulin nephropathy	Nephrotoxicity
Anaphylaxis	Neurotoxicity
Androgen receptor modulation	Non-specific genotoxicity in vitro
Bladder disorders	Non-specific genotoxicity in vivo
Bladder urothelial hyperplasia	Occupational asthma
Blood in urine	Ocular toxicity
Bone marrow toxicity	Oestrogen receptor modulation
Bradycardia	Oestrogenicity
Carcinogenicity	Peroxisome proliferation
Cardiotoxicity	Phospholipidosis
Cerebral oedema	Photo-induced chromosome damage in vitro

Chloracne	Photo-induced non-specific genotoxicity
in vitro	
Cholinesterase inhibition	Photo-induced non-specific genotoxicity
in vivo	
Chromosome damage in vitro	Photoallergenicity
Chromosome damage in vivo	Photocarcinogenicity
Cumulative effect on white cell count and immunology	Photomutagenicity in vitro
Cyanide-type effects	Phototoxicity
Developmental toxicity	Pulmonary toxicity
Glucocorticoid receptor agonism	Respiratory sensitisation
Hepatotoxicity	Skin irritation/corrosion
High acute toxicity	Skin sensitisation HPC
Irritation (of the eye)	Splenotoxicity
Irritation (of the gastrointestinal tract)	Teratogenicity
Irritation (of the respiratory tract)	Testicular toxicity
Kidney disorders	Thyroid toxicity
Kidney function-related toxicity	Uncoupler of oxidative phosphorylation
Lachrymation	Urolithiasis
